# On optimal fuzzy best proximity coincidence points of fuzzy order preserving proximal *Ψ*(*σ*, *α*)-lower-bounding asymptotically contractive mappings in non-Archimedean fuzzy metric spaces

**DOI:** 10.1186/s40064-016-3116-2

**Published:** 2016-09-02

**Authors:** Manuel De la Sen, Mujahid Abbas, Naeem Saleem

**Affiliations:** 1 Faculty of Science and Technology, Institute of Research and Development of Processes IIDP, University of the Basque Country, Barrio Sarriena, P.O. Box 644 de Bilbao, 48940 Leioa, Bizkaia Spain; 2 Department of Mathematics, King Abdulaziz University, P.O. Box 80203, Jidda, 21589 Saudi Arabia; 3 Department of Mathematics and Applied Mathematics, University of Pretoria, Lynnwood Road, Pretoria, 0002 South Africa; 4 Department of Mathematics, National University of Computer and Emerging Sciences, Milaad Street, Lahore, 54000 Pakistan; 5 Department of Mathematics, University of Management and Technology, C-II Johar Town, Lahore, Pakistan

**Keywords:** Fixed points, Best proximity points, Fuzzy set, Fuzzy metric, Optimal fuzzy best proximity coincidence points, Proximal, $\varPsi (\sigma ,\alpha )$-Lower-bounding mapping, $\varPsi (\sigma ,\alpha )$-Lower-bounding asymptotically contractive mapping, Switching rule

## Abstract

This paper discusses some convergence properties in fuzzy ordered proximal approaches defined by $\{ (g_{n} ,T_{n} )\}$—sequences of pairs, where $g:A \to A$ is a surjective self-mapping and $T:A \to B,$ where *A*and *B*are nonempty subsets of and abstract nonempty set *X* and $(X,\,M,\, * ,\,\underline{ \prec } )$ is a partially ordered non-Archimedean fuzzy metric space which is endowed with a fuzzy metric *M*, a triangular norm * and an ordering $\underline{ \prec } .$ The fuzzy set *M* takes values in a sequence or set $\{ M_{{\sigma_{n} }} \}$ where the elements of the so-called switching rule $\{ \sigma_{n} \} \subset \varvec{Z}_{ + }$ are defined from $X \times X \times \varvec{Z}_{0 + }$ to a subset of $\varvec{Z}_{ + } .$ Such a switching rule selects a particular realization of *M* at the *n*th iteration and it is parameterized by a growth evolution sequence $\{ \alpha_{n} \}$ and a sequence or set $\{ \psi_{{\sigma_{n} }} \}$ which belongs to the so-called $\varPsi (\sigma ,\alpha )$-lower-bounding mappings which are defined from [0, 1] to [0, 1]. Some application examples concerning discrete systems under switching rules and best approximation solvability of algebraic equations are discussed.

## Introduction and preliminaries

Results and applications on fuzzy sets in several research disciplines are abundant in the background literature on the field. Related studies have been performed, for instance, in Schweizer and Sklar (1960), George and Veeramani (1994, 1997) and Grabiec (1983), Heilpern (1981), Vetro and Salimi (2013), Gregori and Sapena (1975) and references therein in the context of fuzzy metric spaces since its first introduction and formalization by Zadeh (1965). In particular, a good background on statistical metric spaces is given in Schweizer and Sklar (1960) while some basic results for the analysis of fuzzy metric spaces are given in George and Veeramani (1994, 1997), Gregori and Sapena (1975) including the characterization of fixed points. Among the performed research on the subject, important effort has been devoted to the investigation of the existence and uniqueness of fixed points, best proximity points in cyclic mappings, proximal mappings and partially ordered sets (Vetro and Salimi 2013; Basha 2012; Mongkolkeha et al. 2013; Vetro 2010; De la Sen et al. 2014, 2015; Gabeleh 2015; Rezapour et al. 2011; Al-Thagafi and Shahzad 2009), and also in probabilistic metric spaces (De la Sen and Karapinar 2015; De la Sen and Ibeas 2015, 2016). The study of fuzzy fixed points and its properties has been addressed in Grabiec (1983), Heilpern (1981), Gregori and Sapena (1975), Azam and Beg (2013), De la Sen et al. (2015), Rashid et al. (2015), Chauan et al. (2013b), that of fuzzy best proximity points in De la Sen et al. (2015), those of common fuzzy fixed points in Azam and Beg (2013), Cho et al. (1998), Abbas et al. (2009), Phiangsungnoen et al. (2014), Chauan et al. (2013a, c) and optimal fuzzy best proximity or coincidence points (Basha 2011; Azam and Beg 2013; Cho et al. 1998; Abbas et al. 2009; Phiangsungnoen et al. 2014; Chauan et al. 2013a, c). On the other hand, a detailed research devoted to related properties of convergence of sequences to those relevant points. There are also studies devoted to such topics in classical metric spaces and Banach spaces, not necessarily under the fuzzy formalism, including a lot of research on contractive and non-expansive mappings, self-mappings and, in particular, on cyclic contractive and proximal mappings. See, for instance (Rezapour et al. 2011; Al-Thagafi and Shahzad 2009; Derafshpour et al. 2010; De la Sen and Karapinar 2015; De la Sen et al. 2014), and there are also recent studies on the generalizations and comparison of several types of contractions in Khojasteh et al. (2015) with the introduction of the so-called simulation function. Fixed point theory is also relevant to the stability properties of some iterative schemes or that of dynamic systems (De la Sen and Karapinar 2015; De la Sen and Ibeas 2014, 2015; De la Sen et al. 2015), as an alternative mathematical tool to other classical technique like, for instance, the analysis of Lyapunov stability or hyperstability. See, for instance Se la Sen et al. (2010, 2015), De la Sen (1986), Marchenko (2015a, b). It can be pointed out that recent work in fuzzy metric spaces and probabilistic metric spaces can be found in Grabiec (1983), Cho et al. (1998) and Rashid et al. (2013a, b, c, 2015), respectively. Also, in Khojasteh et al. (2015), the so-called simulation function is introduced and discussed related to a new special generalized contraction which generalizes the Banach contraction and unifies several previously known types of contractions.

There are certain real- life problems for which fixed points, best proximity points, optimal coincidence points or optimal best proximity coincidence points do not exist so that their approximate counterparts are looked for in order to have an approximate solution of the problem at hand. We recall the following basic concepts:

If (*X*, *d*) is a metric space, $A,B \subseteq X$ are nonempty then:

1. *x* $\in$ *A* is a fixed point of $T:A \to A$ if *d*(*x*, *Tx*) = 0,
2. *x*$\in$ *A* is an approximate fixed point of $T:A \to A$ if $d(x,Tx) = inf\{ d(y,Tx):y \in A\} ,$
3. *x* $\in$ *A* is a best proximity point of $T:A \to B$ in *A* if $d(x,Tx) = d(A,B) = \inf \{ d(z,y):z \in A,y \in B\} ,$
4. *x* $\in$ *A* is an approximate best proximity point of $T:A \to B$ in *A* if $d(x,Tx) = inf\{ d(y,Tx):y \in A\} .$

For the case of a pair of two mappings $g:A \to A$ and $T:A \to B$ one has:

(5) *x* $\in$ *A* is an optimal best proximity coincidence point of the pair (*g*, *T*) if $d(gx,Tx) = d(A,B),$
(6) *x* $\in$ *A* is an approximate optimal best proximity coincidence point of the pair (*g*, *T*) if $d(gx,Tx) = inf\{ d(y,Tx):y \in A\} .$

The various above concepts can be extended to the “fuzzy” formalism when dealing with fuzzy metric spaces.

The main objective of this paper is the investigation of some relevant properties of tandems (*g*, *T*) of mappings, where $g:A \to A$ is a non-contractive fuzzy self-mapping and $T:A \to B$ is a fuzzy order preserving proximal so-called *Ψ*(*σ*, *α*)-lower-bounding mapping, where *A*, *B* $\subset$ *X*, $(X,M,\, * ,\,\underline{ \prec } )$ is a partially ordered non-Archimedean fuzzy metric space which is endowed with a fuzzy metric *M*, a triangular norm * (Schweizer and Sklar 1960) and an ordering $\underline{ \prec } ,$ (Basha 2012; Rashid et al. 2015; Saleem et al. 2015). The above function *σ* defined from *A* × *A* to a subset of the natural numbers is the so-called switching law such that each associate sequence {*σ*_*n*_} of its realization is parameterized by a sequence of positive real numbers {*α*_*n*_} which defines a growing condition (contractive, non-expansive or expansive) of the fuzzy set of the form

$$\begin{aligned} & M\left( {x_{n + 2} ,x_{n + 1} ,t} \right) \ge \psi_{{\sigma_{n} }} \left( {M\left( {x_{n + 1} ,x_{n} ,\alpha_{n}^{ - 1} t} \right)} \right) > M\left( {x_{n + 1} ,x_{n} ,\alpha_{n}^{ - 1} t} \right); \\ & \quad \forall n \in \varvec{Z}_{0 + } ,\quad t \in \varvec{R}_{ + } ,\quad \psi_{{\sigma_{n} }} :[0,1] \to [0,1] \\ \end{aligned}$$

belongs to *Ψ*(*σ*, *α*)and $$\{ x_{n} \} \subset A$$ with the values of {*α*_*n*_} remaining constant until a new switching at $$n = n_{i + 1}^{*}$$ occurs, i.e. $$\sigma_{n} = \sigma_{{n_{i}^{*} }} ,$$$$\alpha_{n} = \sigma_{{n_{i}^{*} }} ;$$$$\forall n \in [n_{i}^{*} ,n_{i + 1}^{*} ) \cap \varvec{Z}_{0 + }$$ where $$\{ n_{i}^{*} \} \subset \varvec{Z}_{0 + }$$ is either a strictly sequence (infinite switching) or a finite set (switching with a terminal switching point) of switching points. In particular, the existence, uniqueness and limit properties for proximal sequences of optimal fuzzy best proximity coincidence points is investigated for such pairs of mappings under certain conditions on the switching rules. Note that stability under switched parameterizations is of interest in the fields of dynamic systems since those systems can be driven by different parameterizations through time. See, for instance De la Sen et al. (2015), De la Sen and Ibeas (2016), and references there in. “Optimal fuzzy best proximity coincidence points in partially ordered non-Archimedean fuzzy metric spaces for fuzzy order preserving proximal *Ψ*(*σ*, *α*)-lower-bounding mappings” section is devoted to present, state and prove the formal results. On the other hand, “Examples and associate particular results” section gives three application examples in subjects of stabilization of switched fuzzy discrete dynamic systems and best approximation of resolution of equations in linear algebra and establishes and proves some “ad hoc” specific related results.

### Notation

- ***R*** is the set of real numbers, $\varvec{R}_{ + } = \{ z \in \varvec{R}:z > 0\}$, $\varvec{R}_{0 + } = \varvec{R}_{ + } \cup \{ 0\} ,$
- $${\mathbf{Z}}$$ is the set of integer numbers, $$\varvec{Z}_{ + } = \{ z \in \varvec{Z}:z > 0\} ,$$$$\varvec{Z}_{0 + } = \varvec{Z}_{ + } \cup \{ 0\} ,$$
- $$\bar{p} = \{ 1, \ldots ,p\} ,$$
- $cl( \cdot )$ stands for the closure of the $( \cdot )$-set,

The subsequent equality holds for the t-norm $* :[0,1]^{2} \to [0,1],$

$$\mathop * \limits_{i = m}^{n} M\left( {x,y,t_{i} } \right) = M\left( {x,y_{m} ,t_{m} } \right) * M\left( {y_{m} ,y_{m + 1} ,t_{m + 1} } \right) * \cdots * M\left( {y_{n} ,y,t_{n} } \right),$$

$\left\| {\,A} \right\|_{2} = \sqrt {\lambda_{{max} } (A^{*} A)}$ stands for the ℓ_2_-norm of the complex matrix or vector *A* and $\lambda_{{max} } (A^{*} A)$ denotes the maximum eigenvalue of $A^{*} A.$

Some technical definitions to be then used follow below:

#### **Definition 1** (Schweizer and Sklar 1960)

A binary operation $* :[0,1]^{2} \to [0,1]$ is said to be a continuous t-norm if

(i) * is continuous, commutative and associative,
(ii) $a * 1 = 1$ for all $a \in [0,1],$
(iii) $a * b \le c * d$ if *a* ≤ *c* and *b* ≤ *d*.

The formalism of fuzzy sets was proposed by Zadeh in a general context (Zadeh 1965). The concept of fuzzy metric spaces has been generalized by using continuous *t*-norms in George and Veeramani (1994, 1997). The following formal definition of fuzzy sets on non-Archimedean fuzzy metric spaces will be then used through this manuscript:

#### **Definition 2** (George and Veeramani 1994, 1997)

Let *X* be a nonempty set and * be a continuous t-norm. A fuzzy set *M* on $X \times X \times [0,\infty )$ is said to be a fuzzy metric on the non-Archimedean fuzzy metric space $(X,M, * )$ if for any $x,y,z \in X$, the following conditions hold:

(i) $$M(x,y,t) > 0,$$
(ii) *x* = *y* if and only if $$M(x,y,t) = 1;$$$$\forall t \in \varvec{R}_{ + } ,$$
(iii) $$M(x,y,t) = M(y,x,t),$$
(iv) $$M(x,y,\hbox{max} (t,s)) = M(x,z,t) * M(z,y,s)$$; $$\forall t,s \in \varvec{R}_{ + }$$,
(v) $M(x,y, \cdot ):[0,\infty ) \to [0,1]$ is left-continuous.

If the condition (iv) of Definition 2 is replaced with $$M(x,y,t + s) = M(x,z,t) * M(z,y,s);$$$$\forall t,s \in \varvec{R}_{ + }$$ then $$(X,M, * )$$ is a (Archimedean) fuzzy metric space and $$M(x,y, \cdot )$$ is non-decreasing on $$[0,\infty )$$ and continuous on *X*^2^ × (0, ∞) (Grabiec 1983; Vetro and Salimi 2013; Chauan et al. 2013b; Abbas et al. 2015). If *t* = *s* then $$M(x,y,t) = M(x,z,t) * M(z,y,t)$$; $$\forall t \in \varvec{R}_{ + }$$ and *M* is said to be the strong metric on *X*. Each fuzzy metric *M* on *X* generates a Hausdorff topology *τ*_*M*_ whose base is the family of open balls of members $$B_{M} (x,\varepsilon ,t) = \{ y \in X:M(x,y,t) > 1 - \varepsilon \}$$ for $$\varepsilon \in (0,1),$$$$t \in \varvec{R}_{ + } ,$$ and a sequence $$\{ x_{n} \}$$ converges to *x* $$\in$$ *X* with respect to *τ*_*M*_ if and only if $$lim_{n \to \infty } M(x_{n} ,x,t) = 1$$; $$\forall t \in \varvec{R}_{ + }$$. Note that, since property (iv) implies the above condition, any non-Archimedean fuzzy metric space is a fuzzy metric space.

#### **Definition 3** (Vetro 2010)

Let *A* and *B* be two nonempty subsets of a non-Archimedean fuzzy metric space $(X,M, * )$. Define the sets *A*_0_(*t*) and *B*_0_(*t*) as

$$\begin{aligned} & A_{0} (t) = \left\{ {x \in A:M(x,y,t) = M(A,B,t)\,{\text{for}}\;{\text{some}}\;y \in B} \right\}, \\ & B_{0} (t) = \left\{ {y \in B:M(x,y,t) = M(A,B,t)\,{\text{for}}\;{\text{some}}\;x \in A} \right\} \\ \end{aligned}$$

#### **Definition 4** (Saleem et al. 2015)

Let *Ψ* be the set of all mappings $\psi :[0,1] \to [0,1]$ satisfying the following properties:

(i) *ψ*(0) = 0, *ψ*(1) = 1 and *ψ*(*t*) > *t* for $t \in (0,1)$ and it is continuous in (0, 1),
(ii) $lim_{n \to \infty } \psi^{n} (t) = 1$ if and only if *t* = 1.

The set *Ψ*(*σ*, *α*) used in this paper generalizes the above concept with its elements being functions $\psi_{{\sigma_{n} }}$ parameterized with the sequence of parameterizations of the switching law.

## Optimal fuzzy best proximity coincidence points in partially ordered non-Archimedean fuzzy metric spaces for fuzzy order preserving proximal *Ψ*(*σ*, *α*)-lower-bounding mappings

This section presents, enounces and proves the main formal results which are related to convergence of proximal sequences to existing fuzzy best proximity coincidence points in partially ordered non-Archimedean fuzzy metric spaces $(X,M, * ,\underline{ \prec } )$ for so-called fuzzy order preserving proximal *Ψ*(*σ*, *α*)-lower-bounding mappings where * is a triangular norm, $\underline{ \prec }$ is an ordering relation, *X* is a nonempty set and *M*is a fuzzy set. Such a fuzzy set takes values in a sequence $\{ M_{{\sigma_{n} }} \}$ where the elements of the so-called switching rule $\{ \sigma_{n} \} \subset \varvec{Z}_{ + }$ are defined from $X \times X \times \varvec{Z}_{0 + }$ to a subset of ***Z***_+_.

### **Definition 5**

Let *A* be a nonempty subset of a non-Archimedean fuzzy metric space $(X,M, * )$. See, for instance (Vetro and Salimi 2013; Chauan et al. 2013c; Abbas et al. 2015). A self-mapping *f* on *A* is said to be

1. a fuzzy isometry if $M(fx,fy,t) = M(x,y,t)$ for all $x,y \in A$ and $t \in \varvec{R}_{ + } ,$
2. fuzzy non-contractive if for any $x,y \in A$ and $t \in \varvec{R}_{ + } ,$ we have $M(fx,fy,t) \le M(x,y,t).$

### **Definition 6**

Let $(X,\,\underline{ \prec } )$ be a preordered set and let *A* $\subseteq$ *X* be nonempty sets. A mapping $T:A \to B$ is said to be nondecreasing, or order preserving with respect to a preorder relation $\underline{ \prec }$ on *A*_0_(*t*), if:

- the binary preorder relation $\underline{ \prec }$ on *A* is a partial order relation on *A*_0_(*t*),
- for any *x*, *y* in *A* if *x* $\underline{ \prec }$ *y* then *Tx* $\underline{ \prec }$ *Ty*.

See, for instance Grabiec (1983), Heilpern (1981) for recording some basic concepts on fuzzy mappings and related fixed point theory. The concepts of order preserving, order reversing and monotone mappings $T:A \to B$ have been discussed in Basha (2012), Abbas et al. (2015), where related results have been obtained. An “ad-hoc” adaption of the concept order preserving for the mapping $T:A \to B$ is proposed in the subsequent definitions:

### **Definition 7**

Let $(X,\,\underline{ \prec } )$ and $(X,M,\, * )$ be a preordered set and a non-Archimedean fuzzy metric space, respectively, and let *A*, *B* be nonempty subsets of *X*. A mapping $T:A \to B$ is said to be a fuzzy order preserving proximal *ψ*-contraction if for some $\alpha \in (0,1)$ and any $u,v,x,\,y \in A,$ the following condition holds:

$$\begin{aligned} & \left. {\begin{array}{*{20}l} {x\,\underline{ \prec \,} y} \hfill \\ {M(u,Tx,t) = M\left( {A,B,t} \right)} \hfill \\ {M\left( {v,Ty,t} \right) = M\left( {A,B,t} \right)} \hfill \\ \end{array} } \right\} \Rightarrow \left\{ {\begin{array}{*{20}l} {u\,\underline{ \prec } \,v} \hfill \\ {M\left( {u,v,t} \right) \ge \psi \left( {M\left( {x,y,\alpha^{ - 1} t} \right)} \right),} \hfill \\ \end{array} } \right. \\ & {\text{where}}\,\psi \in \varPsi \quad {\text{for}}\,{\text{all}}\,t \in \varvec{R}_{ + } \\ \end{aligned}$$

If the above conditions holds for any *u*, *v*, *x*, *y* $\in$ *A*_0_(*t*) then $T:A \to B$ is said to be a weak fuzzy order preserving proximal *ψ*-contraction.

The contractions of Definition 7 are sometimes referred to as being contractions of type II (Abbas et al. 2015).

A more general concept than the above contractive one is that of proximal $\varPsi (\sigma ,\alpha )$-lower-bounding mapping (which is non-necessarily contractive) as follows:

### **Definition 8**

Let $(X,\,\underline{ \prec } )$ and $(X,M,\, * )$ be a preordered set and a non-Archimedean fuzzy metric space, respectively, and let *A*, *B* be nonempty subsets of *X*.

1. A mapping $T:A \to B$ is said to be a fuzzy order preserving proximal (or a weak fuzzy order preserving proximal) *Ψ*(*σ*, *α*)-lower-bounding mapping if for any $u,v,x,y \in A_{0} (t)$ the following condition holds:
    $$\left. {\begin{array}{*{20}l} {x\,\underline{ \prec } \,y} \hfill \\ {M(u,Tx,t) = M(A,B,t)} \hfill \\ {M(v,Ty,t) = M(A,B,t)} \hfill \\ \end{array} } \right\} \Rightarrow \left\{ {\begin{array}{*{20}l} {u\,\underline{ \prec } \,v} \hfill \\ {M(u,v,t) \ge \psi \left( {M\left( {x,y,\alpha^{ - 1} t} \right)} \right)} \hfill \\ \end{array} } \right.$$
2. A mapping $T:A \to B$ is said to be a fuzzy order preserving strong proximal *Ψ*(*σ*, *α*)-lower-bounding mapping if for any given sequences $\{ x_{n} \} ,\{ u_{n} \} \subset A$ the following condition holds:
    $$\begin{aligned}& \left. {\begin{array}{*{20}l} {x_{n} \,\underline{ \prec } \,x_{{n + 1_{{}} }} } \hfill \\ {M\left( {u_{n + 1} ,Tx_{n + 1} ,t} \right) \ge M\left( {u_{n} ,Tx_{n} ,t} \right)} \hfill \\ \end{array} } \right\} \\&\quad \Rightarrow \left\{ {\begin{array}{*{20}l} {u_{n} \,\underline{ \prec } \,u_{{n + 1_{{}} }} } \hfill \\ {M\left( {u_{n + 1} ,u_{n} ,t} \right) \ge \psi_{{\sigma_{n} }} \left( {M\left( {x_{n + 1} ,x_{n} ,\alpha^{ - 1} t} \right)} \right)} \hfill \\ {M\left( {u_{n} ,Tx_{n} ,t} \right) \to M\left( {A,B,t} \right)} \hfill \\ \end{array} } \right. \end{aligned}$$
  $$\forall n \in \varvec{Z}_{0 + } ,$$ where $$\psi_{{\sigma_{n} }} \in \varPsi (\sigma ,\alpha )$$ for all $$t \in \varvec{R}_{ + } ,$$$$\alpha = \alpha (x,y,\sigma (x,y,Z_{0 + } )) \in \varvec{R}_{ + }$$ is referred to as the growth evolution rule and $$\sigma :A \times A \times \varvec{Z}_{0 + } \to \bar{p}$$ is referred to as the switching rule, where $$\bar{p} = \{ 1,2, \ldots ,p\} \subset \varvec{Z}_{0 + }$$, and $$\psi \in \varPsi (\sigma ,\alpha )$$.

Note that if *x*_*n*_ = *x*, $x_{n + 1} = y\,(\underline{ \prec } x)$, *u*_*n*+1_ = *u* and *v*_*n*+1_ = *v* are in *A*_0_(*t*) and $M(u,Tx,t) = M(v,Ty,t) = M(A,B,t)$ then $\nu_{n + 1} = \nu(\underline{ \prec } u)$ and $M(u_{n + 1} ,u_{n} ,t) \ge \psi (M(x_{n + 1} ,x_{n} ,\alpha^{ - 1} t)).$ Thus, a strong fuzzy order preserving proximal *Ψ*(*σ*, *α*)-lower-bounding mapping is also a (weak) fuzzy order preserving proximal *Ψ*(*σ*, *α*)-lower-bounding mapping.

In the above definitions, *p* can be finite or infinite, the switching rule can be point-dependent only, i.e. $\sigma = \sigma (x,y),$ it can be iteration-dependent only, i.e. *σ* = *σ*(*n*) for $n \in \varvec{Z}_{0 + }$ when proximal sequences $\{ x_{n} \}$ are built or both, i.e. $\sigma = \sigma (x,y,n)$ for $n \in \varvec{Z}_{0 + }$ (which can be denoted for simplicity by $\sigma_{n} = \sigma_{n} (x,y)$ for $n \in \varvec{Z}_{0 + }$). A particular well-known case is when no switching occurs, i.e. *p* = 1 and $\alpha = \alpha (x,y) \in (0,\bar{\alpha }] \subset (0,1)$. In this case, $T:A \to B$ is said to be a fuzzy order preserving proximal *ψ*-contraction. There is a background literature available on switched maps and applications to stability of dynamic systems and convergence in probabilistic spaces. See, for instance De la Sen and Ibeas (2014, 2015, 2016), De la Sen et al. (2015) and references therein. The concept of best proximity points for non-proximal mappings is discussed in Gabeleh (2015) while giving and proving related results. The problem of common of common fuzzy fixed points in Azam and Beg (2013), Cho et al. (1998), Abbas et al. (2009), Phiangsungnoen et al. (2014), Chauan et al. (2013a, c) for compatible, weakly compatible, non-compatible mappings, and, in Basha (2012) with application of the global minimization of multi-objective functions. Best proximity points are of interest in deterministic, fuzzy and probabilistic frameworks. See, for instance, Mongkolkeha et al. (2013), Vetro (2010), Gabeleh (2015), Rezapour et al. (2011), Al-Thagafi and Shahzad (2009), Derafshpour et al. (2010), De la Sen and Karapinar (2015) and references therein. On the other hand, we have the following definition in the context of fuzzy metric spaces:

### **Definition 9**

A point *z* in an abstract nonempty set *X* is said to be an optimal fuzzy best proximity coincidence point of the pair of mappings $(g,T)$, where $g:A \to A$ is a self-mapping and $T:A \to B$ is, in general, a non-self mapping, *A* and *B* are nonempty subsets of *X* if $M(gz,Tz,t) = M(A,B,t).$

### **Definition 10** (Gregori and Sapena 1975)

A sequence $\{ t_{n} \}$ of positive real numbers is said to be *s*-increasing if there exists $n_{0} \in \varvec{Z}_{0 + }$ such that *t*_*n*+1_ = *t*_*n*_ + 1 for all *n* ≥ *n*_0_.

### **Definition 11** (Vetro and Salimi 2013; Gregori and Sapena 1975)

A fuzzy metric space (*X*, *M*, *) is said to have the property ***T*** if for any *s*-increasing sequence $\{ t_{n} \}$ and any given real constant *ɛ* $\in$ (0, 1), there exists $n_{0} = n_{0} (\varepsilon ) \in \varvec{Z}_{0 + }$ such that $*_{n \ge n_{0}}^{\infty } M(x,y,t_{n} ) \ge 1 - \varepsilon$ for all *n* ≥ *n*_0_ and all $x,y \in X$.

The main result of this section is the subsequent one:

### **Theorem 12**

*Let*$(X,M, * ,\underline{ \prec } )$*be a complete partially ordered non*-*Archimedean fuzzy metric space with the property****T*** and let *A*, *B* $\subseteq$ *X be nonempty sets*; $\forall i \in \bar{p}$*with*$\underline{ \prec }$*being a partial order defined on A*_0_(*t*). *Let a mapping*$T:A \to B$*be continuous and fuzzy order preserving* (*with respect to*$\underline{ \prec }$) *proximal Ψ*(*σ*, *α*)-*lower*-*bounding and let*$g:A \to A$*be surjective, fuzzy non*-*contractive and inverse monotone mapping such that, for any x*, *y* $\in$ *A*, *gx* and *gy are comparable with respect to*$\underline{ \prec }$*only if x*, *y are comparable. Suppose also that each pair of elements of A*_0_(*t*) *has a lower*-*bound and an upper*-*bound, and that for any t* > 0, *A*_0_(*t*) *is nonempty*, *T*(*A*_0_(*t*)) $\subseteq$ *B*_0_(*t*) *and*$A_{0} (t) \subseteq g(A_{0} (t))$. *Assume that for each given x*_0_ $\in$ *A*_0_(*t*) *there exists some element x*_1_*in*$clA_{0\,} (t)$*such that*

1 $$M\left( {gx_{1} ,Tx_{0} ,t} \right) = M(A,B,t)\quad {\text{with}}\;x_{0} \,\underline{ \prec } \,x_{1}$$

Assume also that:

- $$\alpha_{n} = \alpha (z_{n} ,z_{n + 1} ,\sigma_{n} ) \in \varvec{R}_{ + }$$; $$\forall n \in \varvec{Z}_{0 + }$$,
- $$\ell^{ - } M(z_{0} ,z_{1} , + \infty ) = lim_{{t \to \omega^{ - } }} lim_{\omega \to + \infty }\; M(z_{0} ,z_{1} ,t) = 1$$ for any $$z_{0} ,z_{1} \in A_{0} (t),$$
- $$lim_{n \to \infty } \prod\nolimits_{i = 0}^{n} {[\alpha_{i}^{ - 1} (z_{i} ,z_{i + 1} ,\sigma_{i} (z_{i} ,z_{i + 1} ))]} = + \infty$$.

Then, the following properties hold:

(i) There exists z* $\in$ *clA*_0_(*t*) which is an optimal fuzzy best proximity coincidence point of the pair (*g*, *T*) in *A* such that $M(gz^{*} ,Tz^{*} ,t) = M(A,B,t)$ which is the limit of a proximal Cauchy convergent sequence $\{ z_{n} \} \subset A_{0} (t)$.
(ii) Let two distinct proximal sequences $\{ x_{n} \} \subset A_{0} (t)$ and $\{ y_{n} \} \subset A_{0} (t)$ be subject to identical growth evolution rule and switching law $\alpha_{n} = \alpha (x_{n} ,x_{n + 1} ,n) = \alpha (y_{n} ,y_{n + 1} ,n)$ and $\sigma_{n} = \sigma (x_{n} ,x_{n + 1} ,n) = \sigma (y_{n} ,y_{n + 1} ,n)$; $\forall n \in \varvec{Z}_{0 + }$. Then, both sequences are Cauchy convergent sequences to the same limit z* $\in$ *clA*_0_(*t*) which is an optimal fuzzy best proximity coincidence point of the pair (*g*, *T*) in *A*.
(iii) Assume that all the Cauchy proximal sequences in *A*_0_(*t*), being convergent in *clA*_0_(*t*), are subject to identical growth evolution rule and switching law, as it is the case, in particular, if $\alpha_{n} \in \varvec{R}_{ + }$ and $\sigma_{n} \in \bar{p}$ are iteration-dependent (and not point dependent) only. Then, all such Cauchy sequences converge to a unique optimal fuzzy best proximity coincidence point z* $\in$ *clA*_0_(*t*) of the pair (*g*, *T*) in *A*.

### *Proof*

Consider any proximal sequence $\{ z_{n} \} \subset A_{0} (t)$ being defined from some arbitrary given first element *z*_0_ $\in$ *A*_0_(*t*) such that $M(gz_{1} ,Tz_{0} ,t) = M(A,B,t)$ holds for some $z_{1} ( \in A_{0} (t))\,\underline{ \prec } \,z_{0}$. Since $T:A \to B$ be continuous fuzzy order preserving (with respect to $\underline{ \prec }$) proximal $\varPsi (\sigma ,\alpha )$-lower-bounding and $g:A \to A$ is surjective, fuzzy non-contractive and inverse monotone, it follows by contradiction that $z_{1} \,\underline{ \prec } \,z_{2}$. Assume that this is not the case. Since $gz_{1}$ and $gz_{2}$ are comparable what holds, by hypothesis, only if *z*_1_ and *z*_2_ are comparable then $z_{1} \succ z_{2}$ since $z_{1} \,\underline{ \prec } \,z_{2}$ is assumed false. But then $gz_{1} \succ gz_{2}$ which contradicts $gz_{1} \,\underline{ \prec } \,gz_{2}$. Then, $z_{1} \,\underline{ \prec } \,z_{2}$ and, as a result, $z_{0} \,\underline{ \prec } \,z_{1} \,\underline{ \prec } \,z_{2}$. Proceeding in the same way, we can build a sequence $\{ z_{n} \}$ so that the proximal constraint $M(gz_{n + 1} ,Tz_{n} ,t) = M(A,B,t)$ holds for any given $n \in \varvec{Z}_{0 + }$ fulfilling *z*_*n*−1_ $\underline{ \prec }$ *z*_*n*_; $\forall n \in \varvec{Z}_{ + }$ and, furthermore, the set of inequalities:

2 $$\begin{aligned} M\left( {z_{n} ,z_{n + 1} ,t} \right) & \ge M\left( {gz_{n} ,gz_{n + 1} ,t} \right) \\ & \ge \psi_{{\sigma_{n - 1} }} \left( {M\left( {z_{n - 1} ,z_{n} ,\alpha_{n - 1}^{ - 1} t} \right)} \right) \\ & > M\left( {z_{n - 1} ,z_{n} ,\alpha_{n - 1}^{ - 1} t} \right) \\ & \ge \cdots \ge M\left( {z_{0} ,z_{1} ,\prod\limits_{i = 0}^{n - 1} {\left[ {\alpha_{i}^{ - 1} } \right]} t} \right); \\ \end{aligned}$$

$\forall t \in \varvec{R}_{ + }$ where {*σ*_*n*_}and $\{ \alpha_{n} \}$, with $\sigma_{n} = \sigma (z_{n} ,z_{n + 1} ,n) \in \bar{p}$, $\alpha_{n} = \alpha (z_{n} ,z_{n + 1} ,\sigma_{n} ) \in \varvec{R}_{ + }$; $\forall n \in \varvec{Z}_{0 + }$, are the switching and growth evolution sequences, $\psi_{{\sigma_{n} }}$ is in the set *Ψ*(*σ*, *α*); $\forall n \in \varvec{Z}_{0 + } .$ Since $\ell^{ - } M(z_{0} ,z_{1} , + \infty ) = 1$ for any given $z_{0} ,\;z_{1} (\underline{ \prec } z_{0} ) \in A_{0} (t)$, and $lim_{n \to \infty } \prod\nolimits_{i = 0}^{n} {[\alpha_{i}^{ - 1} (z_{i} ,z_{i + 1} ,\sigma_{i} (z_{i} ,z_{i + 1} ))]} = + \infty$, then it follows from (2) and the property ***T***:

3 $$\mathop {lim}\limits_{{\omega \to t^{ - } }} \mathop {lim}\limits_{n \to \infty } M\left( {z_{n} ,z_{n + 1} ,\omega } \right) = 1 ;\quad \forall t \in \varvec{R}_{ + }$$

Thus, for any given *ɛ* $\in$ (0, 1), there exists $N = N(\varepsilon ) \in \varvec{Z}_{0 + }$ such that $M(z_{n} ,z_{n + 1} ,t) > 1 - \varepsilon$ and $z_{n} \,\underline{ \prec } \,z_{n + 1}$ for any $n( \ge N) \in \varvec{Z}_{0 + }$ and then $\{ z_{n} \} \subset A_{0} (t)$ is a Cauchy sequence for the given first element *z*_0_ $\in$ *A*_0_(*t*). Such a sequence is convergent to some $(X,M, * ,\underline{ \prec } )$ since $(X,M, * ,\underline{ \prec } )$ is complete. Since $M(z_{0} ,z_{1} ,\prod\nolimits_{i = 0}^{n - 1} {[\alpha_{i}^{ - 1} ]} t) \le M(gz_{n} ,gz_{n + 1} ,t) \le M\left( {z_{n} ,z_{n + 1} ,t} \right)$; $\forall t \in \varvec{R}_{ + }$, $\{ z_{n} \} \to z^{*}$, *T* is continuous and then one gets from (3)

4 $$\begin{aligned} 1 & = \mathop {lim}\limits_{{\omega \to t^{ - } }} \mathop {lim}\limits_{n \to \infty } M\left( {z_{0} ,z_{1} ,\prod\limits_{i = 0}^{n - 1} {\left[ {\alpha_{i}^{ - 1}} \right] \omega } } \right) \le \mathop {lim}\limits_{n \to \infty } M\left( {gz_{n} ,gz_{n + 1} ,t} \right) \\ & \le \mathop {lim}\limits_{n \to \infty } M\left( {z_{n} ,z_{n + 1} ,t} \right) = \mathop {lim}\limits_{{\omega \to t^{ - } }} \mathop {lim}\limits_{n \to \infty } M\left( {z^{*} ,z^{*} ,t} \right) = 1; \\ \end{aligned}$$

$\forall t \in \varvec{R}_{ + }$ and then z* $\in$ *clA*_0_(*t*) which is an optimal fuzzy best proximity coincidence point of the pair (*g*, *T*) since $\{ Tz_{n} \} \to Tz^{*}$ since $T:A \to B$ is continuous and $\{ Tz_{n} \} \to Tz^{*}$, and

5 $$M\left( {gz^{*} ,Tz^{*} ,t} \right) = \mathop {lim}\limits_{n \to \infty } M\left( {gz_{n + 1} ,Tz_{n} ,t} \right) = M\left( {A,B,t} \right);$$

$\forall t \in \varvec{R}_{ + }$, $\forall n \in \varvec{Z}_{0 + }$. Property (i) has been proved.

(ii) The fact that both proximal sequences are Cauchy and convergent to optimal fuzzy best proximity coincidence points of the pair (*g*, *T*) in *A* follows from Property (i). It is now proved that both have the same limit under the same growth evolution rule and switching law. Assume, on the contrary, that there are two points $x^{*} ,y^{*} ( \ne x^{*} ) \in clA_{0} (t)$ such that there are two distinct convergent Cauchy sequences $\{ x_{n} \} \to x^{*}$ and $\{ y_{n} \} \to y^{*} .$ Thus, for any given $\varepsilon \in (0,1)$ there exist $N_{x} = N_{x} (\varepsilon ),N_{y} = N_{y} (\varepsilon ) \in \varvec{Z}_{0 + }$ such that
  $$\hbox{min} \left( {M\left( {x_{n} ,x_{n + 1} ,t} \right),M\left( {y_{n} ,y_{n + 1} ,t} \right)} \right) > 1 - \varepsilon ;\quad \forall n( \ge N) \in \varvec{Z}_{0 + }$$
  where *N* = *N*(*ɛ*) = max(*N*_*x*_, *N*_*y*_), and
  $$\begin{aligned} & M\left( {x_{n} ,x^{*} ,t} \right) \ge M\left( {x_{n} ,x_{n + 1} ,t} \right) * M\left( {x_{n + 1} ,x^{*} ,t} \right);\quad \forall t \in \varvec{R}_{ + } \\ & M\left( {y_{n} ,y^{*} ,t} \right) \ge M\left( {y_{n} ,y_{n + 1} ,t} \right) * M\left( {y_{n + 1} ,y^{*} ,t} \right);\quad \forall t \in \varvec{R}_{ + } \\ \end{aligned}$$
  so that
  $$\mathop {lim}\limits_{n \to \infty } M\left( {x_{n} ,x^{*} ,t} \right) = \mathop {lim}\limits_{n \to \infty } M\left( {y_{n} ,y^{*} ,t} \right) = 1;\quad \forall t \in \varvec{R}_{ + }$$
  with $x_{n + 1} \,\underline{ \prec \,} x_{n} \,\underline{ \prec } \,x^{*}$ and $y_{n + 1} \,\underline{ \prec } \,y_{n} \,\underline{ \prec } \,y^{*}$ for $n \in \varvec{Z}_{0 + }$. Then$\hbox{min} (M(x_{m} ,x^{*} ,t),M(y_{m} ,y^{*} ,t)) > 1 - \varepsilon$; $\forall m( \ge M) \in \varvec{Z}_{0 + }$; $\forall t \in {\mathbf{R}}_{ + }$ and some $M = M(\varepsilon )( \ge N) \in \varvec{Z}_{0 + }$, and,

### *Case a*

if x* and y* are comparable then

6 $$\begin{aligned} M\left( {x^{*} ,y^{*} ,t} \right) & \ge \left( {M\left( {x_{n + 1} ,x^{*} ,t} \right) * M\left( {y^{*} ,y_{n + 1} ,t} \right)} \right) * M\left( {x_{n + 1} ,y_{n + 1} ,t} \right) \\ & \ge \left( {M\left( {x_{n + 1} ,x^{*} ,t} \right) * M\left( {y^{*} ,y_{n + 1} ,t} \right)} \right) \\ & \quad * M\left( {x_{n + 1} ,y_{n + 1} ,t} \right) \\ & \ge \left( {M\left( {x_{n + 1} ,x^{*} ,t} \right) * M\left( {y^{*} ,y_{n + 1} ,t} \right)} \right) \\ & \quad * M\left( {gx_{n + 1} ,gy_{n + 1} ,t} \right) \\ & \ge \left( {M\left( {x_{n + 1} ,x^{*} ,t} \right) * M\left( {y^{*} ,y_{n + 1} ,t} \right)} \right) \\ & \quad * \psi_{{\sigma_{n} }} \left( {M\left( {x_{n} ,y_{n} ,\alpha_{n}^{ - 1} t} \right)} \right) \\ & > \left( {M\left( {x_{n + 1} ,x^{*} ,t} \right) * M\left( {y^{*} ,y_{n + 1} ,t} \right)} \right) \\ & \quad *M\left( {x_{n} ,y_{n} ,\alpha_{n}^{ - 1} t} \right) \\ & \ge \cdots \ge \left( {M\left( {x_{n + 1} ,x^{*} ,t} \right) * M\left( {y^{*} ,y_{n + 1} ,t} \right)} \right) \\ &\quad *M\left( {x_{0} ,y_{1} ,\prod\limits_{i = 0}^{n - 1} {\left[ {\alpha_{i}^{ - 1} } \right]t} } \right) \\ & \ge \left( {\left( {1 - \varepsilon } \right) * \left( {1 - \varepsilon } \right)} \right) * \left( {1 - \varepsilon } \right);\quad \forall t \in \varvec{R}_{ + } ,\quad \forall n( \ge N^{*} ) \in \varvec{Z}_{0 + } \\ \end{aligned}$$

with *N* * = N*(*ɛ*) = *M* + *N*_2_ = *N* + *N*_1_ + *N*_2_ and some $$N_{1} = N_{1} (\varepsilon ),N_{2} = N_{2} (\varepsilon ) \in \varvec{Z}_{0 + }$$. Then, since *ɛ* $$\in$$ (0, 1) is arbitrary, $$lim_{{\omega \to t^{ - } }} M(x^{*} ,y^{*} ,\omega ) = 1;$$$$\forall t \in \varvec{R}_{ + }$$ which implies that x* = y*, provided they are comparable, a contradiction. Thus, any proximal sequence is a convergent Cauchy sequence all of them converging to the same unique limit $$z^{*} \in clA_{0} (t)$$ provided that they are built with the same growth evolution rule and the same switching law.

### *Case b*

In the case that there exist two non-comparable distinct limits $x^{*} ,y^{*} ( \ne x^{*} ) \in clA_{0} (t)$ for two proximal Cauchy convergent sequences, then it follows by contradiction also that x* = y* from the assumption that $g:A \to A$ is inverse monotone. Assume that x* ≠ y*. Thus, the convergent sequences $\{ x_{n} \}$ and $\{ y_{n} \}$ are lower-bounded and upper-bounded by the same sequences $\{ u_{n} \}$ and $\{ \bar{u}_{n} \} ,$ respectively, which are both of them bounded and totally ordered, of comparable terms and convergent, that is $u_{n + 1} \,\underline{ \prec } \,u_{n} \,\underline{ \prec } \,u^{*}$ and $\bar{u}_{n + 1} \,\underline{ \prec } \,\bar{u}_{n} \,\underline{ \prec } \,\bar{u}^{*}$ for all $n \in \varvec{Z}_{0 + }$. Then, from thee above case for comparable limits, u*and $\bar{u}^{*}$ are mutually comparable, then identical, and comparable as well to x^*^and y^*^so that $u^{*} \,\underline{ \prec } \,x^{*} \,\underline{ \prec } \,u^{*}$ and $u^{*} \,\underline{ \prec } \,y^{*} \,\underline{ \prec } \,u^{*}$ since $\bar{u}^{*} = u^{*} .$ Then, it follows that x* = y*. Property (i) has been proved. Property (iii) is a direct consequence of Property (ii). □

### **Definition 13**

Let *A* and *B* be two nonempty subsets of a non-Archimedean fuzzy metric space $(X,M, * )$. The set *B* is said to be fuzzy approximatively compact with respect to *A* if each sequence $\{ Tx_{n} \} \subset B$ such that $\{ M(x,Tx_{n} ,t)\} \to M(x,B,t)$ for some *x* $\in$ *A* has a convergent subsequence.

We can get the subsequent Corollary to Theorem 12:

### **Corollary 14**

Theorem 12 *holds* “*mutatis*-*mutandis*” *if B is fuzzy approximatively compact with respect to A* (*even if *$T:A \to B$*is not everywhere continuous*).

### *Proof*

The hypothesis of Theorem 12 still hold except that $T:A \to B$ is not necessarily everywhere continuous while *B* is fuzzy approximatively compact with respect to *A*. Then, the first part of the proof of Theorem 12 is still applicable while one concludes that $\{ z_{n} \} \to z^{*}$ is a convergent Cauchy sequence and that $\{ gz_{n} \} \to gz^{*}$ for any chosen proximal sequence so that there is a convergent subsequence $\{ Tz_{{n_{k} }} \} ( \subset \{ Tz_{n} \} \subset clA_{0} (t)) \to \bar{z}^{*}$ as *k* → ∞ for some $z^{*} \in clA_{0} (t)$ since

7 $$\begin{aligned} M\left( {gz^{*} ,Tz^{*} ,t} \right) & \ge \mathop {lim\,inf}\limits_{k \to \infty } M\left( {gz^{*} ,Tz_{{n_{k} }} ,t} \right) \\ & = \mathop {lim}\limits_{k \to \infty } M\left( {gz_{{n_{k} }} ,\bar{z}^{*} ,t} \right) \\ & = \mathop {lim}\limits_{k \to \infty } M\left( {gz^{*} ,\bar{z}^{*} ,t} \right) \\ & = \mathop {lim}\limits_{k \to \infty } M\left( {gz^{*} ,\bar{z}^{*} ,t} \right) \\ & = M(A,B,t) \\ \end{aligned}$$

leads to

8 $$\begin{aligned} & \left( {M\left( {gz_{n} Tz_{n} ,t} \right) - M\left( {gz^{*} ,Tz_{{n_{k} }} ,t} \right)} \right) \to 0, \\ & \quad M\left( {gz^{*} ,Tz,t} \right) \to M\left( {gz^{*} ,B,t} \right) = M\left( {A,B,t} \right) \\ \end{aligned}$$

as $k,n \to \infty$ (thus, *n*_*k*_ → ∞) and then $\bar{z}^{*} = Tz^{*} .$ Assume the contrary $\bar{z}^{*} \ne Tz^{*}$ so that one gets:

$$\begin{aligned} & 1 > M\left( {\bar{z}^{*} ,Tz^{*} ,t} \right) \ge \left( {M\left( {\bar{z}^{*} ,Tz_{{n_{k} }} ,t} \right) * M\left( {Tz_{{n_{k} }} ,Tz_{n} ,t} \right)} \right) * M\left( {Tz_{n} ,Tz^{*} ,t} \right); \\ & \quad \quad \forall t \in \varvec{R}_{ + } ,\quad \forall n \in \varvec{Z}_{0 + } \\ \end{aligned}$$

what leads to the subsequent contradiction since $\{ Tz_{{n_{k} }} \} \to \bar{z}^{*}$, $(\{ Tz_{n} \} - \{ Tz_{{n_{k} }} \} ) \to 0$ and $\{ z_{n} \} \to z^{*}$:

9 $$\begin{aligned} & 1 > \left( {\mathop {lim}\limits_{k \to \infty } M\left( {\bar{z}^{*} ,Tz_{{n_{k} }} ,t} \right) * \mathop {lim}\limits_{k,n \to \infty } M\left( {Tz_{{n_{k} }} ,Tz_{n} ,t} \right)} \right) * \mathop {lim}\limits_{n \to \infty } M\left( {Tz_{n} ,Tz^{*} ,t} \right) \\ & \quad = \left( {1 * 1} \right) * 1 = 1 \\ \end{aligned}$$

Thus, $\bar{z}^{*} = Tz^{*}$ and z* $\in$ *clA*_0_(*t*) which is an optimal fuzzy best proximity coincidence point of the pair (*g*, *T*) in *A* such that $M(gz^{*} ,Tz^{*} ,t) = M(A,B,t)$ which is the limit of a proximal Cauchy convergent sequence $\{ z_{n} \} \subset A_{0} (t)$. This proves Property (i) of Theorem 12 if the everywhere continuity condition of $T:A \to B$ is replaced with that of fuzzy approximative compactness. Properties [(ii)–(iii)] of Theorem 12 can be also proved under the condition replacing the continuity of $T:A \to B$. □

Theorem 12 and Corollary 14 also hold if $T:A \to B$ is a continuous fuzzy order preserving (with respect to $\underline{ \prec }$) proximal $\varPsi (\sigma ,\alpha )$-lower-bounding mapping if constructed subsequences in the whole *A* converge asymptotically to be proximal subsequences (asymptotically convergent proximal sequences) converging to a unique optimal fuzzy best proximity coincidence point of (*g*, *T*) (Vetro and Salimi 2013; Abbas et al. 2015) in *A* so that $M(gz_{n + 1} ,Tz_{n} ,t) \to M(A,B\,,t)$ as *n* → ∞. This can be set up more rigorously as follows.

### **Definition 15**

Let

(1) $(X,M, * ,\underline{ \prec } )$ be a complete partially ordered non-Archimedean fuzzy metric space, $g:A \to A$,

(2) $T:A \to B$ be a fuzzy order preserving (with respect to $\underline{ \prec }$) strong proximal $\varPsi (\sigma ,\alpha )$-lower-bounding mapping (Definition 8.2).

A Cauchy sequence $\{ z_{n} \} \subset A$ is said to be an asymptotically convergent proximal sequence with respect to (*g*, *T*) if $z_{n + 1} \,\underline{ \prec } \,z_{n}$; $\forall n \in \varvec{Z}_{0 + }$, $\{ z_{n} \} \to z^{*}$ and $\{ M(gz_{n + 1} ,Tz_{n} )\} \to M(A,B,t) = M(gz^{*} ,Tz^{*} ,t)$.

Note that the limit z^*^of $\{ z_{n} \}$ is an optimal fuzzy best proximity coincidence point of (*g*, *T*). Note also that the asymptotically convergent proximal sequence can have points in *A* which are not necessarily in $clA_{0} (t)$.

If, furthermore, $T:A \to B$ is continuous (or, alternatively, if *B*is fuzzy approximatively compact with respect to *A*) and $g:A \to A$ is surjective, fuzzy non-contractive and inverse monotone mapping such that, for any *x*, *y* $\in$ *A*, *gx* and *gx* are comparable with respect to $\underline{ \prec }$ only if *x*, *y* are comparable then the following extensions of Theorem 12 and its counterpart of Corollary 14 follow:

### **Theorem 16**

*Let*$(X,M, * ,\underline{ \prec } )$*be a complete partially ordered non*-*Archimedean fuzzy metric space with the property****T*** and let *A*, *B* $\subseteq$ *X be nonempty sets with*$\underline{ \prec }$*being a partial order defined on A*_0_(*t*). *Let a mapping*$T :\,A \to B$*be continuous and fuzzy order preserving* [*with respect to*$\underline{ \prec }$ (Basha 2012)] *strong proximal Ψ*(*σ*, *α*)-*lower*-*bounding and let*$g:A \to A$*be surjective, fuzzy non*-*contractive and inverse monotone mapping such that, for any x*, *y* $\in$ *A*, *gx* and *gy are comparable with respect to*$\underline{ \prec }$*only if x*, *y are comparable. Suppose also that each pair of elements of A*_0_(*t*) *has a lower*-*bound and an upper*-*bound, and that for any t* > 0, *A*_0_(*t*) *is nonempty, T*(*A*_0_(*t*)) $\subseteq$ *B*_0_(*t*) *and*$A_{0} (t) \subseteq g(A_{0} (t))$. *Assume that for each given x*_0_ $\in$ *A*_0_(*t*) *there exist some element x*_1_*in*$clA_{0\,} (t)$*such that*

10 $$M(gx_{1} ,Tx_{0} ,t) = M(A,B,t)\quad {\text{with}}\; x_{0} \,\underline{ \prec } \,x_{1}$$

Assume also that:

- $$\alpha_{n} = \alpha (z_{n} ,z_{n + 1} ,\sigma_{n} ) \in \varvec{R}_{ + }$$; $$\forall n \in \varvec{Z}_{0 + },$$
- $$\ell^{ - } M(z_{0} ,z_{1} , + \infty ) = lim_{{\omega \to t^{ - } }} lim_{t \to + \infty } M(z_{0} ,z_{1} ,t) = 1$$ for any $$z_{0} ,z_{1} \in A_{0} (t),$$
- $$lim_{n \to \infty } \prod\nolimits_{i = 0}^{n} {\left[ {\alpha_{i}^{ - 1} (z_{i} ,z_{i + 1} ,\sigma_{i} (z_{i} ,z_{i + 1} ))} \right]} = + \infty$$

Then, the following properties hold:

(i) There exists $z^{*} \in clA_{0} (t)$ which is an optimal fuzzy best proximity coincidence point of the pair (*g*, *T*) in *A* such that $M(gz^{*} ,Tz^{*} ,t) = M(A,B,t)$ which is the limit of some sequence $\{ z_{n} \}$ which is asymptotically convergent proximal Cauchy with respect to the pair (*g*, *T*),
(ii) Let two distinct proximal sequences $\{ x_{n} \} \subset A$ and $\{ y_{n} \} \subset A$ be subject to identical growth evolution rule and switching law $\alpha_{n} = \alpha (x_{n} ,x_{n + 1} ,n) = \alpha (y_{n} ,y_{n + 1} ,n)$ and $\sigma_{n} = \sigma (x_{n} ,x_{n + 1} ,n) = \sigma (y_{n} ,y_{n + 1} ,n)$; $\forall n \in \varvec{Z}_{0 + }$. Then, both sequences are asymptotically convergent proximal Cauchy sequences to the same limit z* $\in$ *clA*_0_(*t*) which is an optimal fuzzy best proximity coincidence point of the pair (*g*, *T*) in *A*,
(iii) Assume that all the asymptotically convergent proximal Cauchy sequences in *A*, being convergent in *clA*_0_(*t*), are subject to identical growth evolution rule and switching law, as it is the case, in particular, if $\alpha_{n} \in \varvec{R}_{ + }$ and $\sigma_{n} \in \bar{p}$ are iteration-dependent (and not point dependent) only. Then, all such Cauchy sequences converge to a unique optimal fuzzy best proximity coincidence point z* $\in$ *clA*_0_(*t*) of the pair (*g*, *T*) in*A*.

### *Sketch of proof*

Now take *z*_0_ $\in$ *A*(arbitrary) and some existing $z_{1} (\underline{ \prec } z_{0} ),z_{n + 1} (\underline{ \prec } z_{n} )$ such that {*z*_*n*_} $\subset$ *A*. $\{ Tz_{n} \} \subset B$ and $M(gz_{n + 1} ,Tz_{n} ,\alpha_{n}^{ - 1} t) \le M(gz_{n + 2} ,Tz_{n + 1} ,t) \le M(A,B,t)$; $\forall n \in \varvec{Z}_{0 + }$. Since $T:A \to B$ is a fuzzy order preserving strong proximal $\varPsi (\sigma ,\alpha )$-lower-bounding mapping and $g:A \to B$ is surjective, fuzzy non-contractive and inverse monotone mapping such that, for any *x*, *y* $\in$ *A*, *gx* and *gy* are comparable with respect to $\underline{ \prec }$ only if *x*, *y* are comparable, one has for any built subsequence $z_{n} \in \{ z_{n} \} \subset A$ of the whole iterative process, since $\psi :[0,1] \to [0,1]$ is in the set *Ψ*, and one gets that (2)–(4) and (6) still hold, but now $\{ z_{n} \} \subset A$, and also

11 $$\begin{aligned} M\left( {A,B,t} \right) & \ge M\left( {z_{n} ,z_{n + 1} ,t} \right) \ge M\left( {gz_{n} ,gz_{n + 1} ,t} \right) \\ & \ge \psi \left( {M\left( {z_{n - 1} ,z_{n} ,\alpha_{n - 1}^{ - 1} t} \right)} \right) > M\left( {z_{n - 1} ,z_{n} ,\alpha_{n - 1}^{ - 1} t} \right);\quad \forall n \in \varvec{Z}_{0 + } \\ \end{aligned}$$

One can conclude from the steps of the proof of Theorem 12 and from the sketch of proof of Corollary 14 that $M(z_{n - 1} ,z_{n} ,t) \to M(A,B,t)$ as *n* → ∞so that the subsequence *z*_*n*_ converges to a best proximity point in *cl*(*A*_0_(*t*)) which is an optimal fuzzy best proximity coincidence point of (*g*, *T*)in*A*, that is, $\{ M(gz_{n + 1} ,Tz_{n} ,t)\} \to M(A,B,t) = M(gz^{*} ,Tz^{*} ,t)$. This proves Property (i) while Properties [(ii)–(iii)] follow under the added assumptions as their counterparts of Theorem 12. □

### **Corollary 17**

Theorem 16 *also holds for asymptotically convergent proximal sequences with respect to* (*g*, *T*) if *B is fuzzy approximatively compact with respect to A* (*even if*$T:A \to B$*is not everywhere continuous*).

The following result, which is a consequence of Theorem 16 and Corollary 17, is of interest for its use in real situations:

### **Corollary 18**

*Let*$(X,M, * ,\underline{ \prec } )$*be a complete partially ordered non*-*Archimedean fuzzy metric space and let A*, *B* $\subseteq$ *X be nonempty sets with*$\underline{ \prec }$*being a partial order defined on A*_0_(*t*), *such that the following conditions hold*:

1. *Either*$(X,M, * ,\underline{ \prec } )$*possesses the property****T****or, alternatively*, $M(x,y, \cdot ):[0,\infty ) \times [\theta ,\infty ) \to [0,1]$*is strictly increasing for some*$\theta \in \varvec{R}_{0 + }$,
2. *The mapping*$g:A \to A$*is surjective, fuzzy non*-*contractive and inverse monotone such that, for any x*, *y* $\in$ *A*, *gx* and *gy are comparable with respect to*$\underline{ \prec }$*only if x*, *y are comparable*,
3. *Each pair of elements of A*_0_(*t*) *has a lower*-*bound and an upper*-*bound, and for any t* > 0, *A*_0_(*t*) *is nonempty, T*(*A*_0_(*t*)) $\subseteq$ *B*_0_(*t*) *and*$A_{0} (t) \subseteq g(A_{0} (t))$*and also, for each given z*_0_ $\in$ *A*_0_(*t*), *there exist some element z*_1_*in*$clA_{0\,} (t)$*such that*$M(gz_{1} ,Tz_{0} ,t) = M(A,B,t)$*with*$z_{0} \,\underline{ \prec } \,z_{1} ,$*and*$\ell^{ - } M(z_{0} ,z_{1} , + \infty ) = 1$*for any*$z_{0} ,z_{1} \in A_{0} (t),$
4. *The mapping*$T:A \to B$*is fuzzy order preserving strong proximal Ψ*(*σ*, *α*)-*lower*-*bounding, such that any sequence*$\{ z_{n} \} \subset A$*built accordingly to*$M(gz_{n + 2} ,Tz_{n + 1} ,t) \ge M(gz_{n + 1} ,Tz_{n} ,\alpha_{n}^{ - 1} t)$; $\forall n \in \varvec{Z}_{0 + }$, $\forall t \in \varvec{R}_{ + }$, *and subject to a switching law and associate growth evolution rule defined by*:
  12 $$\begin{aligned} & \sigma_{n} = \sigma_{n} (z_{n} ,z_{n + 1} ,n) = \sigma_{{n_{i}^{*} }} = \sigma_{j} = \sigma_{{j\left( {n_{i}^{*} } \right)}} \quad {\text{for}}\,{\text{all}}\,n\left( { \in \varvec{Z}_{0 + } } \right) \in \left[ {n_{i}^{*} ,n_{i + 1}^{*} } \right) \\ & \alpha_{n} = \alpha_{n} (z_{n} ,z_{n + 1} ,n) = \alpha_{{n_{i}^{*} }} = \alpha_{j} = \alpha_{{j\left( {n_{i}^{*} } \right)}} , \\ & \forall n\left( { \in \varvec{Z}_{0 + } } \right) \in \left[ {n_{i}^{*} ,n_{i + 1}^{*} } \right),\quad \forall i \in \varvec{Z}_{0 + } \,{\text{which}}\,{\text{satisfy}}\,{\text{the}}\,{\text{constraint:}} \\ & \prod\limits_{j = 0}^{i} {\left[ {\alpha_{{n_{j}^{*} }}^{{n_{j + 1}^{*} }} } \right]} \alpha_{{n_{i + 1}^{*} }}^{{n_{i + 2}^{*} - n_{i + 1}^{*} }} < 1;\quad \forall i \in \varvec{Z}_{0 + } \\ \end{aligned}$$
  *with*$$\alpha_{0}^{{n_{1}^{*} }} < \frac{1}{{\alpha_{{n_{i}^{*} }}^{{n_{i + 1}^{*} - n_{i}^{*} }} }}$$*for some*$$j = j(n_{i}^{*} ) \in \bar{p}$$: (a) *either strictly increasing sequence*$$\{ n_{i}^{*} \} \subseteq \varvec{Z}_{0 + }$$, or (b) *some finite strictly ordered set of nonnegative integers with*$$\bar{\ell } = card\{ n_{i}^{*} :i = 1,2, \ldots ,\ell \}$$ and *α*_ℓ_ $$\in$$ (0, 1), *with*$$n_{0}^{*} = 0$$ and $$(n_{i + 1}^{*} - n_{i}^{*} ) \le N < \infty ;$$$$\forall i \in \varvec{Z}_{0 + }$$, *of switching points, that is*$$\sigma_{{n_{i + 1}^{*} }} \ne \sigma_{{n_{i}^{*} }} ,$$*with*$$\sigma_{{n_{i}^{*} }} ,\sigma_{{n_{i + 1}^{*} }} \in \bar{p},$$*and*$$\alpha_{{n_{i + 1}^{*} }} = \alpha_{{\sigma (n_{i + 1}^{*} )}} \ne \alpha_{{n_{i}^{*} }} = \alpha_{{\sigma (n_{i}^{*} )}}$$; $$\forall i \in \varvec{Z}_{0 + }$$.

Then, Theorem 16 holds if $T:A \to B$ is continuous and Corollary 17 holds if *B* is fuzzy approximatively compact with respect to *A*.

### *Outline of the proof*

The proof follows from the fact that *n*_0_^*^ = 0, and

- if $\{ n_{i}^{*} \} \subseteq \varvec{Z}_{0 + }$ is a strictly increasing sequence (i.e. there are infinitely many switches), then
  13 $$\begin{aligned} & \mathop {lim\;sup}\limits_{n \to \infty } \prod\limits_{i = 0}^{n} {\left[ {\alpha_{i}^{ - 1} \left( {z_{i} ,z_{i + 1} ,\sigma_{i} \left( {z_{i} ,z_{i + 1} } \right)} \right)} \right]} \\ & \quad = \mathop {lim}\limits_{i \to \infty } \prod\limits_{j = 0}^{i} {\left[ {\alpha_{{n_{j}^{*} }}^{{ - \left( {n_{j + 1}^{*} - n_{j}^{*} } \right)}} \left( {z_{j} ,z_{j + 1} ,\sigma_{j} \left( {z_{j} ,z_{j + 1} } \right)} \right)} \right]} = + \infty \\ \end{aligned}$$
  since:
  $$\prod\limits_{j = 0}^{i} {\left[ {\alpha_{{n_{j}^{*} }}^{{n_{j + 1}^{*} }} } \right]} < 1;\quad \forall i \in \varvec{Z}_{0}^{ + } \quad {\text{and}}\quad \mathop {lim}\limits_{i \to \infty } \prod\limits_{j = 0}^{i} {\left[ {\alpha_{{n_{j}^{*} }}^{{n_{j + 1}^{*} }} } \right]} < 1$$
- if there is a finite number of ℓ switches and $\alpha_{\ell } \in (0,1)$ then
  $$\prod\limits_{j = 0}^{i} {\left[ {\alpha_{{n_{j}^{*} }}^{{n_{j + 1}^{*} }} } \right]} < 1\quad {\text{for}}\,i \in \bar{\ell } = card\left\{ {n_{i}^{*} :i = 1,2, \ldots ,\ell } \right\}$$
  so that $lim_{n \to \infty } \prod\nolimits_{i = 0}^{n} {[\alpha_{i}^{ - 1} (z_{i} ,z_{i + 1} ,\sigma_{i} (z_{i} ,z_{i + 1} ))]} = + \infty$. Then from Theorem 16, or, eventually, from Corollary 17, it follows $\{ z_{n} \} \to z^{*}$ and, furthermore, $\{ M(gz_{n + 1} ,Tz_{n} ,t)\} \to M(A,B,t) = M(gz^{*} ,Tz^{*} ,t)$ for some $z^{*} \in clA_{0} \left( t \right)$.□

### **Corollary 19**

Corollary 18 *holds, under the same assumptions, if* (12) *is replaced with*:

14 $$\alpha_{0}^{{n_{1}^{*} }} < \frac{1}{{\alpha_{{n_{i}^{*} }}^{{n_{i + 1}^{*} - n_{i}^{*} }} }},\quad \alpha_{{n_{i + 1}^{*} }}^{{n_{i + 2}^{*} - n_{i + 1}^{*} }} < \frac{1}{{\alpha_{{n_{i}^{*} }}^{{n_{i + 1}^{*} - n_{i}^{*} }} }};\quad \forall i \in \varvec{Z}_{0 + }$$

### *Sketch of proof*

Note that (13) guarantees that (12) holds for all $i \in \varvec{Z}_{0 + }$ with $n_{0}^{*} = 0.$ □

### **Corollary 20**

Corollaries 18 and 19 *also hold, under the same assumptions, if there is some strictly increasing sequence of marked switching points*$\{ n_{i*}^{*} \} \subseteq \{ n_{i}^{*} \} ,$*with first element*$n_{0*}^{*} = n_{0}^{*} = 0,$*fulfilling the following condition instead of* (12):

15 $$\prod\limits_{{j = n_{{i^{*} }} }}^{{n_{{\left( {i + 1} \right)^{*} }} - 1}} {\left[ {\alpha_{{n_{j}^{*} }}^{{n_{j + 1}^{*} }} } \right] < 1} \quad {\text{for}}\,{\text{all}}\,n_{i*}^{*} \in \left\{ {n_{i*}^{*} } \right\}$$

### *Sketch of proof*

Note from (14) that

16 $$\prod\limits_{{j = n_{{i^{*} }} }}^{{n_{{\left( {i + 1} \right)^{*} }} - 1}} {\left[ {\alpha_{{n_{j}^{*} }}^{{n_{j + 1}^{*} }} } \right] = \prod\limits_{{j \in J\left( {i^{*} ,(i + 1)^{*} } \right),i \in \varvec{Z}_{0 + } }} {\left[ {\alpha_{{n_{j}^{*} }}^{{n_{j + 1}^{*} - n_{j}^{*} }} } \right] < 1} } \quad {\text{for}}\,{\text{all}}\,n_{i*}^{*} \in \left\{ {n_{i*}^{*} } \right\}$$

where *card*$J(i^{*} ,(i + 1)^{*} ) = n_{{\left( {i + 1} \right)^{*} }}^{*} - n_{{i^{*} }}^{*} = \sum\nolimits_{{j = i^{*} }}^{{(i + 1)^{*} - 1}} {n_{j + 1}^{*} - n_{j}^{*} }$. Then, it follows as in the proof of Corollary 16 that

$$\mathop {lim\;sup}\limits_{n \to \infty } \prod\limits_{i = 0}^{n} {\left[ {\alpha_{i}^{ - 1} \left( {z_{i} ,z_{i + 1} ,\sigma_{i} \left( {z_{i} ,z_{i + 1} } \right)} \right)} \right]} = \mathop {lim}\limits_{i \to \infty } \prod\limits_{j = 0}^{i} {\left[ {\alpha_{{n_{j}^{*} }}^{{ - \left( {n_{j + 1}^{*} - n_{j}^{*} } \right)}} \left( {z_{j} ,z_{j + 1} ,\sigma_{j} \left( {z_{j} ,z_{j + 1} } \right)} \right)} \right] = + \infty }$$

□

### *Remark 21*

Note that *p*, such that ∞ ≥ *p* ≥ 1, in Corollary 18 is the number of available configurations for switching, i.e. the “switching” is reflected in the inequalities related to the mapping $M:A \times A \times [0,\infty ) \to [0,1]$ for an asymptotically convergent proximal sequence $\{ z_{n} \} \subset A$ which converge to an optimal fuzzy best proximity coincidence point in *A*_0_(*t*), under the hypotheses of Corollary 18, as follows:

17 $$\begin{aligned} M\left( {z_{n + 2} ,z_{n + 1} ,t} \right) & \ge M\left( {gz_{n + 2} ,gz_{n + 1} ,t} \right) \ge \psi_{{\sigma_{{n_{i}^{*} }} }} \left( {M\left( {z_{n + 1} ,z_{n} ,\alpha_{{n_{i}^{*} }}^{ - 1} t} \right)} \right) \\ & > M\left( {z_{n + 1} ,z_{n} ,\alpha_{{n_{i}^{*} }}^{ - 1} t} \right) \ne M\left( {z_{n + 1} ,z_{n} ,\alpha_{n - 1}^{ - 1} t} \right)\quad {\text{if}}\,n = n_{i}^{*} \quad {\text{for}}\,{\text{any}}\,{\text{given}}\,i \in \varvec{Z}_{0 + } \\ M\left( {z_{n + 2} ,z_{n + 1} ,t} \right) & \ge M\left( {gz_{n + 2} ,gz_{n + 1} ,t} \right) \ge \psi_{{\sigma_{{n_{i - 1}^{*} }} }} \left( {M\left( {z_{n + 1} ,z_{n} ,\alpha_{n}^{ - 1} t} \right)} \right) \\ & > M\left( {z_{n + 1} ,z_{n} ,\alpha_{n}^{ - 1} t} \right) = M\left( {z_{n + 1} ,z_{n} ,\alpha_{{n_{i - 1}^{*} }}^{ - 1} t} \right)\quad {\text{if}}\,n \in \left[ {n_{i - 1}^{*} ,n_{i}^{*} } \right)\quad {\text{for}}\,{\text{any}}\,{\text{given}}\,i \in \varvec{Z}_{ + } . \\ \end{aligned}$$

### *Remark 22*

Note that for some given *p* such that ∞ ≥ *p* ≥ 1,

$$\sigma_{n} \in \bar{p} = \{ 1,2, \ldots ,p\} ,\quad \alpha_{n} \in \varOmega = \left\{ {\alpha_{1} ,\alpha_{2} , \ldots ,\alpha_{p} } \right\};\quad \forall n \in \varvec{Z}_{0 + }$$

Thus, it turns out that a necessary condition for Corollary 18 to hold is that $\alpha_{n} \in (0,1)$ for at least some $n \in \bar{p}$. It turns also out that if the switching law is such that in the case when *S* = {*n*_i_^*^} is a strictly ordered finite set (i.e. only a finite number of switches is performed) with $n_{max}^{*} = max\{ n \in S\}$, and in this case necessarily *p* < ∞, then $\alpha_{{n_{max}^{*} }} \in (0,1).$

## Examples and associate particular results

Three worked examples are given in this section which are related to two useful applications related, in particular, to stabilization of switched fuzzy discrete dynamic systems and to best approximation of resolution of equations in linear algebra. Some “ad hoc” specific related results are also established and proved.

### *Example 23*

$(X,M, * ,\underline{ \prec } )$ be an ordered set and a non-Archimedean fuzzy metric space (Vetro and Salimi 2013; Chauan et al. 2013b; Abbas et al. 2015) endowed with a fuzzy metric $M_{{\sigma_{n} }} \left( {x,y,\alpha_{n} } \right) = \frac{t}{t + d(x,y)}$; $\forall x,y \in X$, $\forall t \in \varvec{R}_{ + }$, where $d:X \times X \to \varvec{R}_{0 + }$ is the metric of the Banach space $(X,\left\| \cdot \right\|)$, being also a complete metric space $(X,d)$, the distance being identified with the norm, where:

1. $$X = A = B = \varvec{R},$$
2. $$\sigma_{n} \in \bar{p} = \{ 1,2, \ldots ,p\}$$ is a numerable set of switching laws for some $$p \in {\mathbf{Z}}_{ + }$$ with parameterizations $$\alpha_{n} \in \varOmega = \{ \alpha_{1} ,\alpha_{2} , \ldots ,\alpha_{p} \} \subset \varvec{R}_{ + }$$ (see Remark 22), $$\psi_{{\sigma_{n} }} \in \varPsi$$ is defined by $$\psi_{{\sigma_{n} }} (t) = t^{{1/m(\sigma_{n} )}} ;$$$$\forall n \in \varvec{Z}_{0 + }$$ where $$m(\sigma_{n} ) \in \varvec{Z}_{ + }$$; $$\forall n \in \varvec{Z}_{0 + }$$
3. $T:\varvec{R} \to \varvec{R}$ is a fuzzy order preserving strong proximal $\varPsi (\sigma ,\alpha )$-lower-bounding mapping which, together with the non-contractive invertible mapping $g:\varvec{R} \to \varvec{R}$, describes the solution of the positive discrete *n*th dimensional linear system as follows:
    18 $$G_{n} x_{n + 1} = A_{n} x_{n} + v_{n} ;\quad \forall n \in \varvec{Z}_{0 + }$$
    for any initial condition $x_{0} = a \in \varvec{R}^{n}$, *v*_*n*_ = *Q*_*n*_*x*_*n*_; $\forall n \in \varvec{Z}_{0 + }$ quantifies the unmodeled dynamics (De la Sen et al. 2010; De la Sen 1986; Marchenko 2015a, b), and $A_{n} ,G_{n} ,Q_{n} \in \varvec{R}^{n \times n}$; $\forall n \in \varvec{Z}_{0 + }$. This implies that:
    19 $$G_{n} x_{n + 1} = \left( {A_{n} + Q_{n} } \right)x_{n} ;\quad \forall n \in \varvec{Z}_{0 + }$$
    provided that *G*_*n*_ is nonsingular; $\forall n \in \varvec{Z}_{0 + }$. If *I* denotes the *n*-th identity matrix then
    20 $$\left\| {x_{n + 1} - x_{n} } \right\| = \left\| {\left( {G_{n}^{ - 1} \left( {A_{n} + Q_{n} } \right) - I} \right)x_{n} } \right\|;\quad \forall n \in \varvec{Z}_{0 + }$$

The condition (11) holds if:

21 $$\begin{aligned} \frac{t}{{t + \left\| {\left( {G_{n}^{ - 1} \left( {A_{n} + Q_{n} } \right) - I} \right)x_{n} } \right\|}} & \ge \left( {\frac{t}{{t + \alpha_{n} \left\| {\left( {G_{n - 1}^{ - 1} \left( {A_{n - 1} + Q_{n - 1} } \right) - I} \right)x_{n - 1} } \right\|}}} \right)^{{1/m_{n} }} \\ & > \frac{t}{{t + \alpha_{n} \left\| {\left( {G_{n - 1}^{ - 1} \left( {A_{n - 1} + Q_{n - 1} } \right) - I} \right)x_{n - 1} } \right\|}};\quad \forall n \in \varvec{Z}_{ + } \\ \end{aligned}$$

where $m_{n} = m_{{(\sigma_{n - \ell \left( n \right)} )}}$ and *α*_*n*_ = *α*(*σ*_*n*−ℓ(*n*)_); $\forall n \in \varvec{Z}_{0 + }$ for some $\ell (n) \in \varvec{Z}_{0 + }$. Note that if ℓ(*n*) = 0, then there is a switching at the *n*th iteration so that either *α*_*n*_ = *α*(*σ*_*n*_) ≠ *α*_*n*−1_ = *α*(*σ*_*n*−1_) or *m*_*n*_ = *m*(*σ*_*n*_) ≠ *m*_*n*−1_ = *m*(*σ*_*n*−1_). Note that at least one of the two values should be distinct from its value at the preceding iteration step if there is a switching at the *n*-th iteration so that $\sigma_{n} ( \ne \sigma_{n - 1} ) \in \bar{p}$

22 $$\begin{aligned} & \left\| {\left( {G_{n}^{ - 1} \left( {A_{n} + Q_{n} } \right) - I} \right)x_{n} } \right\| \le \left( {t + \alpha_{n} \left\| {\left( {G_{n - 1}^{ - 1} \left( {A_{n - 1} + Q_{n - 1} } \right) - I} \right)x_{n - 1} } \right\|} \right)^{{1/m_{n} }} t^{{\left( {m_{n} - 1} \right)/m_{n} }} - t; \\ & \quad \forall n \in \varvec{Z}_{ + } ,\quad \forall t \in \varvec{R}_{ + } , \\ \end{aligned}$$

or, using (19) to calculate *x*_*n*_:

23 $$\alpha_{n} \ge \frac{{\left( {\left( {\left\| {\left( {G_{n}^{ - 1} \left( {A_{n} + Q_{n} } \right) - I} \right)G_{n - 1}^{ - 1} \left( {A_{n - 1} + Q_{n - 1} } \right)x_{n - 1} } \right\| + t} \right)t^{{ - m_{n} }} - 1} \right)t}}{{\left\| {\left( {G_{n - 1}^{ - 1} \left( {A_{n - 1} + Q_{n - 1} } \right) - I} \right)x_{n - 1} } \right\|}};\quad \forall n \in \varvec{Z}_{ + } ;\quad t \in \varvec{R}_{ + }$$

Since *m*_*n*_ ≥ 1, the lower-bound of (23) is zero at *t* = 0 and decreasing to (−∞) in ***R***_+_ for any $n \in \varvec{Z}_{0 + }$. Then, it is satisfied for any nonnegative real sequence $\{ \alpha_{n} \}$. Note that

- if *m*_*n*_ = 1, which leads to the use of the last right-hand-side lower-bounding in (21) irrespective of the particular used functions in the set $\varPsi (\sigma ,\alpha )$, then (23) becomes:
  24 $$\alpha_{n} \ge \frac{{\left\| {M_{n} R_{n - 1} x_{n - 1} } \right\|}}{{\left\| {M_{n - 1} x_{n - 1} } \right\|}} = \left( {\frac{{x_{n - 1}^{T} R_{n - 1}^{T} M_{n}^{T} M_{n} R_{n - 1} x_{n - 1} }}{{x_{n - 1}^{T} M_{n - 1}^{T} M_{n - 1} x_{n - 1} }}} \right)^{{^{{^{1/2} }} }}$$
  where the superscript *T* denotes matrix and vector transposition and, if *λ*_max_(·)and *λ*_min_(·) denote, respectively, the maximum and minimum eigenvalue of the symmetric (·)-matrix and *λ*(·) denotes some eigenvalue of the (·)-matrix, then
  $$M_{n} = G_{n}^{ - 1} \left( {A_{n} + Q_{n} } \right) - I;\quad R_{n} = G_{n}^{ - 1} \left( {A_{n} + Q_{n} } \right) = M_{n} + I;$$
  and
  25 $$\begin{aligned} \alpha_{n} & \ge \sqrt {\frac{{\lambda_{{max} } \left( {R_{n - 1}^{T} M_{n}^{T} M_{n} R_{n - 1} } \right)^{{}} }}{{\lambda_{{min} } \left( {M_{n - 1}^{T} M_{n - 1} } \right)^{{}} }}} \\ & = \frac{{\left| {\lambda \left( {M_{n} R_{n - 1} } \right)} \right|_{{max} } }}{{\left| {\lambda \left( {M_{n - 1} } \right)} \right|_{{min} } }} = \frac{{\left| {\lambda \left( {M_{n} \left( {M_{n - 1} + I} \right)} \right)} \right|_{{max} } }}{{\left| {\lambda \left( {M_{n - 1} } \right)} \right|_{{min} } }} \\ \end{aligned}$$
  is a sufficient condition for (24) to hold.
- if *m*_*n*_ > 1, then $\psi_{{\sigma_{n} }} (z) = z^{{1/m\left( {\sigma_{n} } \right)}} > z$ for $z \in (0,1)$ leads to the same conclusion and (25) is a sufficient condition for (23) to hold for all $t \in \varvec{R}_{ + }$. Then (11) also holds if (25) holds, i.e. $M(x_{n} ,x_{n + 1} ,t) > M(x_{n - 1} ,x_{n} ,\alpha_{n - 1}^{ - 1} t)$; $\forall n \in \varvec{Z}_{ + }$, $\forall t \in \varvec{R}_{ + }$. Thus, one gets the following result:

### **Proposition 24**

*Assume that, for any given*$x_{0} = a \in \varvec{R}^{n}$:

(A) *If*$e_{i}^{T} (G_{n - 1}^{ - 1} (A_{n - 1} + Q_{n - 1} ) - I)x_{n - 1} \ne 0$*then, for each*$i \in \bar{p}$*and*$n \in \varvec{Z}_{ + } ,$*either*
  $$\text{sgn} \left[ {e_{i}^{T} \left( {G_{n}^{ - 1} \left( {A_{n} + Q_{n} } \right) - I} \right)x_{n} } \right] = \text{sgn} \left[ {e_{i}^{T} \left( {G_{n - 1}^{ - 1} \left( {A_{n - 1} + Q_{n - 1} } \right) - I} \right)x_{n - 1} } \right];\quad \forall i \in \bar{p},$$
  *or*$$e_{i}^{T} (G_{n}^{ - 1} (A_{n} + Q_{n} ) - I)x_{n} = 0,$$
  (b) $$e_{i}^{T} (G_{n - 1}^{ - 1} (A_{n - 1} + Q_{n - 1} ) - I)x_{n - 1} = 0 \Rightarrow e_{i}^{T} (G_{n}^{ - 1} \left( {A + Q_{n} } \right) - I)x_{n} = 0$$
    *where e*_*i*_*is the i*th *unity vector in the canonical basis of****R***^*n*^.
(B) *Assume also that a switching law*$\sigma :\varvec{R}^{2} \times \varvec{Z}_{0 + } \to \bar{p}$*with a growth evolution rule*$\alpha_{n} = \alpha (x_{n + 1} ,x_{n} ,\sigma (x_{n + 1} ,x_{n} ,Z_{0 + } )) \in \varOmega$*which satisfies* (25) and
  26 $$\mathop {lim}\limits_{n \to \infty } \prod\limits_{i = 0}^{n} {\left[ {\alpha_{i}^{ - 1} \left( {x_{i} ,x_{i + 1} ,\sigma_{i} \left( {x_{i} ,x_{i + 1} } \right)} \right)} \right] = + \infty }$$

The constraint (26) is guaranteed, in particular, if the switching rule obeys the initial constraint $\alpha_{0}^{{n_{1}^{*} }} < \frac{1}{{\alpha_{{n_{i}^{*} }}^{{n_{i + 1}^{*} - n_{i}^{*} }} }}$ and some of the constraints below which guarantee that (26) holds:

27 $$(1)\,\,\prod\limits_{j = 0}^{i} {\left[ {\alpha_{{n_{j}^{*} }}^{{n_{j + 1}^{*} }} } \right]\alpha_{{n_{i + 1}^{*} }}^{{n_{i + 2}^{*} - n_{i + 1}^{*} }} < 1} ,$$

28 $$(2)\,\,\alpha_{{n_{i + 1}^{*} }}^{{n_{i + 2}^{*} - n_{i + 1}^{*} }} < \frac{1}{{\alpha_{{n_{i}^{*} }}^{{n_{i + 1}^{*} - n_{i}^{*} }} }};\quad \forall i \in \varvec{Z}_{0 + } ,$$

29 $$(3)\,\,\prod\limits_{{j = n_{{i^{*} }} }}^{{n_{{\left( {i + 1} \right)^{*} }} - 1}} {\left[ {\alpha_{{n_{j}^{*} }}^{{n_{j + 1}^{*} }} } \right] < 1\quad {\text{for}}\,{\text{all}}\,n_{i*}^{*} \; \in \left\{ {n_{{i^{*} }}^{*} } \right\}\left( { \subseteq \left\{ {n_{i}^{*} } \right\}} \right)}$$

where $\{ n_{i}^{*} \}$ and $\{ n_{i*}^{*} \} \subseteq \{ n_{i}^{*} \}$ are, respectively, the sets of switching points and marked set of switching points. Then, $\mathop {lim}\nolimits_{n \to \infty } M(x_{n} ,x_{n + 1} ,t) = 1$ and $\{ x_{n} \}$ converges.

### *Proof*

The given first assumption (a) together with the constraints (25), or (23), guarantees a component-wise ordering and also that $T:\varvec{R} \to \varvec{R}$ is a fuzzy order preserving strong proximal $\varPsi (\sigma ,\alpha )$-lower-bounding mapping. If the switching law, with its associate growth rule, satisfies (26) or, in particular any of the constraints (27)–(29), then, $\mathop {lim}\nolimits_{n \to \infty } M(x_{n} ,x_{n + 1} ,t) = 1$ and $\{ x_{n} \}$ converges from Theorem 16 and Corollaries 18–20. □

The following particular result of Proposition 24 for Example 23 is relevant in practical cases for this problem under conditions of positivity and decreasing conditions of the solution.

### *Example 25*

A particular case of interest of Example 23/Proposition 24 follows below under conditions of positivity of the solutions (i.e. $A = B = \varvec{R}_{0 + }^{n}$) being strictly decreasing to a zero equilibrium point.

### **Proposition 26**

*The following properties hold:*

(i) *Assume that the entries of the sequences of matrices* {*A*_*n*_} and {*Q*_*n*_} *satisfy the entry*-*per*-*entry constraint*$$(Q_{n} )_{ij} \ge - (A_{n} )_{ij} ;$$$$\forall i,j \in \bar{n}$$, $$\forall n \in \varvec{Z}_{0 + }$$*and that the sequence* {*G*_*n*_} *consist of monomial (or generalized permutation) matrices with its nonzero entries being positive. Then,*$$\{ G_{n}^{ - 1} (A_{n} + Q_{n} )\} \subset \varvec{R}_{0 + }^{n \times n}$$.
(ii) *Assume that the sequences*$\{ (A_{n} + Q_{n} )\}$*and*$\{ (A_{n} + Q_{n} )^{ - 1} G_{n} \}$*are, respectively, nonsingular and consist of monomial matrices. Then,*$\{ G_{n}^{ - 1} (A_{n} + Q_{n} )\} \subset \varvec{R}_{0 + }^{n \times n}$.
(iii) *Assume that*$$a \in \varvec{R}_{0 + }^{n}$$, $$\{ G_{n}^{ - 1} (A_{n} + Q_{n} )\} \subset \varvec{R}_{0 + }^{n \times n}$$ (*equivalently if*$$(A_{n} + Q_{n} )^{ - 1} G_{n}$$*and it is a monomial matrix) and*$$1 > \lambda_{\hbox{max} } (((A_{n}^{T} + Q_{n}^{T} )G_{n}^{ - T} G_{n}^{ - 1} (A_{n} + Q_{n} )));$$$$\forall n \in \varvec{Z}_{0 + }$$*and that the* assumption B of Proposition 24 *holds for a switching law*$$\sigma :\varvec{R}^{2} \times \varvec{Z}_{0 + } \to \bar{p}$$*with a growth evolution rule*$$\alpha_{n} = \alpha (x_{n + 1} ,x_{n} ,\sigma (x_{n + 1} ,x_{n} ,\varvec{Z}_{0 + } )) \in \varOmega$$. *Then,*$$lim_{n \to \infty } M(x_{n} ,x_{n + 1} ,t) = 1$$ and $$\{ x_{n} \} \to 0$$.

### *Proof*

If $$(Q_{n} )_{ij} \ge - (A_{n} )_{ij} ;$$$$\forall i,j \in \bar{n},$$$$\forall n \in \varvec{Z}_{0 + }$$ then $$\{ (A_{n} + Q_{n} )\} \subset \varvec{R}_{0 + }^{n \times n}$$ and if $$G_{n} \in \varvec{R}{}^{n \times n}$$; $$\forall n \in \varvec{Z}_{0 + }$$ is monomial then $$\{ G_{n}^{ - 1} \} \subset \varvec{R}_{0 + }^{n \times n}$$. As a result, direct calculus via matrix multiplication yields $$G_{n}^{ - 1} (A_{n} + Q_{n} ) \in \varvec{R}_{0 + }^{n \times n}$$; $$\forall n \in \varvec{Z}_{0 + }$$. Property (i) has been proved. If $$(A_{n} + Q_{n} )^{ - 1} G_{n}$$; $$\forall n \in \varvec{Z}_{0 + }$$ exists and is monomial then $$G_{n}^{ - 1} (A_{n} + Q_{n} ) \in \varvec{R}_{0 + }^{n \times n}$$ (since the inverse of a positive matrix is positive if and only if such a matrix is monomial); $$\forall n \in \varvec{Z}_{0 + }$$ and Property (ii) follows.

Now, note that:

30 $$- \tilde{x}_{n} = x_{n} - x_{n + 1} = \left( {I - G_{n}^{ - 1} (A_{n} + Q_{n} )} \right)x_{n} ;\quad \forall n \in \varvec{Z}_{0 + }$$

has all its components nonnegative for any $n \in \varvec{Z}_{0 + }$ if $\{ x_{n} \} \subset \varvec{R}_{0 + }^{n}$ and $\{ I - G_{n}^{ - 1} (A_{n} + Q_{n} )\} \subset \varvec{R}_{0 + }^{n \times n}$ (then $x_{n + 1} \,\underline{ \prec } \,x_{n}$, with the ordering “$\underline{ \prec }$” (Basha 2011, 2012), being defined as component-wise “≤”-ordering, and $\{ G_{n}^{ - 1} (A_{n} + Q_{n} )\}$ is a sequence of convergent matrices; $\forall n \in \varvec{Z}_{0 + }$) which is guaranteed if $1 > \lambda_{\hbox{max} } (((A_{n}^{T} + Q_{n}^{T} )G_{n}^{ - T} G_{n}^{ - 1} (A_{n} + Q_{n} )))$; $\forall n \in \varvec{Z}_{0 + }$. On the other hand, $\{ x_{n} \} \subset \varvec{R}_{0 + }^{n}$ if $a \in \varvec{R}_{0 + }^{n}$ and $G_{n}^{ - 1} (A_{n} + Q_{n} ) \in \varvec{R}_{0 + }^{n \times n}$. Since $x_{n + 1} \,\underline{ \prec } \,x_{n}$; $\forall n \in \varvec{Z}_{0 + }$, $a \in \varvec{R}_{0 + }^{n}$ and $\{ x_{n} \} \subset \varvec{R}_{0 + }^{n}$, under the assumptions of Property (iii), then $\mathop {lim}\nolimits_{n \to \infty } M(x_{n} ,x_{n + 1} ,t) = 1$ and $\{ x_{n} \} \to 0$. □

### *Example 27* (Generalized matrix inversion to calculate optimal approximate solutions)

Define the mappings *g*and *T* by respective matrix sequences $\{ G_{n} \} \in \varvec{C}^{s \times q}$ and, $\{ L_{n} \} \in \varvec{C}^{s \times q}$ and $\{ B_{n} \} \in \varvec{C}^{s \times r}$, with *s* ≤ *q*, to solve either exactly or approximately solved the proximal constraint in *X*_*n*+1_ for any given *X*_*n*_ and any $n \in \varvec{Z}_{0}$ with $\{ X_{n} \} \subset \varvec{C}^{q \times r}$:

31 $$G_{n} X_{n + 1} = L_{n} X_{n} + B_{n} ;\quad \forall n \in \varvec{Z}_{0 + }$$

It is well-known from the Rouché–Froebenius theorem from linear algebra that for the case of constant matrices *G*_*n*_ = *G*, *L*_*n*_ = *L*, *B*_*n*_ = *B*, *X*_*n*_ = *X*; $\forall n \in \varvec{Z}_{0 + }$, there are infinitely many solutions (compatible indeterminate system) if $rank(G - L) = rank(G - L,B) \le \hbox{min} (q,s) = s$ and a unique one $X = (G - L)^{ - 1} B$ if the above rank equality holds with $rank(G - L) = q = s$. If the system is compatible indeterminate or if it is incompatible [i.e. $rank(G - L) < rank(G - L,B) \le s = min(q,s)$] then best approximations to the solutions can be computed in the sense of minimizing $\left\| {GX - LX - B} \right\|$ in *X* for some prefixed matrix norm. The approximated solutions through an iterative process in (31) is stated so as to solve the next iteration either exactly or approximately in $\{ X_{n} \}$:

32 $$G_{n} X_{n + 1} = L_{n} X_{n} + B_{n} ;\quad \forall n \in \varvec{Z}_{0 + }$$

Let $G_{n}^{\dag }$ be the generalized inverse (or pseudoinverse) of*G*_*n*_. If $G_{n} \in \varvec{C}^{s \times q}$ is of rank *r*_*n*_ ≤ *s* then there exist $C_{n} \in \varvec{C}^{{s \times r_{n} }}$ and $D_{n} \in \varvec{C}^{{r_{n} \times q}}$ such that the following factorization exists $G_{n} = C_{n} D_{n}$; $\forall n \in \varvec{Z}_{0 + }$ and $G_{n}^{{{\dag }}} = D_{n}^{*} (D_{n} D_{n}^{*} )^{ - 1} (C_{n}^{*} C_{n} )^{ - 1} C_{n}^{*}$; $\forall n \in \varvec{Z}_{0 + }$, where the superscript “*” stands for the complex conjugate, is the so-called Moore–Penrose generalized inverse (Barnett 1971; Moore 1935; De la Sen et al. 2014). Any solution sequence {*X*_*n*_} of (32) is given by

33 $$X_{n + 1} = G_{n}^{{{\dag }}} \left( {L_{n} X_{n} + B_{n} } \right) + \left( {I - G_{n}^{{{\dag }}} G_{n} } \right)Y_{n} ;\quad \forall n \in \varvec{Z}_{0 + } ,$$

where *I* is the *q*th identity matrix, for any given arbitrary sequence $\{ Y_{n} \} \subset \varvec{C}^{q \times r}$ provided that the following necessary and sufficient condition for a solution to exist holds:

34 $$\left( {I - G_{n} G_{n}^{{{\dag }}} } \right)\left( {L_{n} X_{n} + B_{n} } \right) = 0;\quad \forall n \in \varvec{Z}_{0 + }$$

It is known that for any $n \in \varvec{Z}_{0 + }$ and any given $X_{n} \in \varvec{C}^{q \times r}$ the best approximation solution (33) of (32) is obtained if *Y*_*n*_ = 0, i.e.

35 $$\begin{aligned} X_{n + 1}^{0} & = G_{n}^{\dag } \left( {L_{n} X_{n} + B_{n} } \right) \\ & = Arg\left( {X_{n + 1} \in \varvec{C}^{q \times r} :\left\| {G_{n} X_{n + 1} - L_{n} X_{n} - B_{n} } \right\| < \left\| {G_{n} \bar{X}_{n + 1} - L_{n} X_{n} - B_{n} } \right\|,\quad \forall \bar{X}_{n + 1} \in C^{q \times r} } \right) \\ \end{aligned}$$

### **Theorem 28**

*Assume that there exist sequences*$$\{ \tilde{L}_{n} \} \subset \varvec{C}^{s \times q}$$*and*$$\{ \tilde{M}_{n} \} \subset \varvec{C}^{s \times q}$$*such that*$$B_{n} = \tilde{L}_{n} X_{n}$$ and $$Y_{n} = \tilde{M}_{n} X_{n} ;$$$$\forall n \in \varvec{Z}_{0 + }$$. *Then, the following properties hold:*

(i) *Define the matrix sequence*$\{ H_{n} \} \subset \varvec{C}^{q \times q}$*of elements:*
  36 $$\begin{aligned} H_{n} & = \left[ {\left( {L_{n}^{*} G_{n}^{{*^{{{\dag }}} }} + G_{n}^{{{\dag }}} L_{n} } \right) + \left( {\tilde{L}_{n}^{*} G_{n}^{{*^{{{\dag }}} }} + \tilde{M}_{n}^{*} \left( {I - G_{n}^{*} G_{n}^{{*^{{{\dag }}} }} } \right)} \right)\left( {G_{n}^{{{\dag }}} \tilde{L}_{n} + \left( {I - G_{n}^{{{\dag }}} G_{n} } \right)\tilde{M}_{n} } \right)} \right. \\ & \quad \left. { +\, L_{n}^{*} G_{n}^{{*^{{{\dag }}} }} \left( {G_{n}^{{{\dag }}} \tilde{L}_{n} + \left( {I - G_{n}^{{{\dag }}} G_{n} } \right)\tilde{M}_{n} } \right) + \left( {\tilde{L}_{n}^{*} G_{n}^{{*^{{{\dag }}} }} + \tilde{M}_{n}^{*} \left( {I - G_{n}^{*} G_{n}^{{*^{{{\dag }}} }} } \right)} \right)G_{n}^{{{\dag }}} L_{n} } \right];\quad \forall n \in \varvec{Z}_{0 + } \\ \end{aligned}$$
  *If either*$$lim\,sup_{n \to \infty } \left\| {H_{n} } \right\|_{2} \le 1$$*or if*$$\sum\nolimits_{i = 0}^{\infty } {(I - H_{n} )}$$*is bounded and positive semidefinite then the solution iteration sequence* {*X*_*n*_} *of* (33) *fulfils*$$lim_{n \to \infty } X_{n}^{*} X_{n} = \bar{X} = \hat{X}^{*} \hat{X}$$*with*$$\left\| {X_{n}^{*} X_{n} } \right\| = \left\| {X_{n} } \right\|_{2}^{2} < \infty;$$$$\forall n \in \varvec{Z}_{0 + }$$*and*$$\left\| {\hat{X}} \right\|_{2} < \infty$$.
(ii) *Consider the best approximation solution*$X^{0} = (G - L)^{{{\dag }}} B$*solution nominal algebraic equation*$GX = LX + B.$*Then the solution to the iterative scheme* (31) *withG*_*n*_ = *G*, *L*_*n*_ = *LandB*_*n*_ = *B*; $\forall n \in \varvec{Z}_{0 + }$*converges toX*^0^*if*
  - $$\left\| {G^{{^{{{\dag }}} }} L} \right\|_{2} < 1$$
  - *the involved G*, *L and Bmatrices are real and G is full row rank* (*i.e. it is associated with a surjective mapping *) and $- (G^{{^{{{\dag }}} }} L) \ge 0$ (*i.e.*$G^{{^{{{\dag }}} }} L$*has no positive entry*).
(iii) *Assume that all the conditions of* Property (i) *hold and consider the iterative scheme* (31) *with* {*G*_*n*_} → *G*, $$\{ L_{n} \} \to L$$*and*$$\{ B_{n} \} \to B.$$*Then,*$$lim\,sup_{n \to \infty } \left\| {X_{n}^{0} - X^{0} } \right\| \le \frac{K\delta }{1 - \rho }$$*where*$$K \in \varvec{R}_{ + },$$$$\rho \in (0,1)$$*and*$$\delta = o\left[sup_{{n \in Z_{0 + } }} \left(\left\| {\tilde{B}_{n} } \right\|_{2} + \left\| {\tilde{L}_{n} } \right\|_{2} + \left\| {\tilde{G}_{n} } \right\|_{2} \right)\right]$$.
(iv) *Assume that G*_*n*_*is full row rank, i.e.*$$rank\,G_{n} = s;$$$$\forall n \in \varvec{Z}_{0 + }$$*there is a switching law*$$\sigma :\varvec{R}^{2} \times \varvec{Z}_{0 + } \to \bar{p}$$*with a growth evolution rule*$$\alpha_{n} = \alpha (x_{n + 1} ,x_{n} ,\sigma (x_{n + 1} ,x_{n} ,Z_{0 + } )) \in \varOmega,$$*under control functions*$$\psi_{{\sigma_{n} }} \in \varPsi (\sigma ,\alpha );$$$$\forall n \in \varvec{Z}_{0 + },$$*such that*
  37 $$\alpha_{n} \ge \frac{{\left( {\left( {\left\| {\left( {G_{n + 1}^{{{\dag }}} L_{n + 1} - I} \right)\left( {G_{n}^{{{\dag }}} \left( {L_{n} X_{n}^{0} + B_{n} } \right)} \right) + G_{n + 1}^{{{\dag }}} B_{n + 1} } \right\|_{2} + t} \right)t^{{ - m_{n} }} - 1} \right)t}}{{\left\| {\left( { \, G_{n}^{{{\dag }}} L_{n} - I} \right)X_{n}^{0} + G_{n}^{{{\dag }}} B_{n} } \right\|_{2} }};\quad \forall n \in \varvec{Z}_{0 + } ;\quad t \in \varvec{R}_{ + }$$
  *for some sequence*$\{ m_{n} \} \subset \varvec{Z}_{ + },$*and*
  38 $$\mathop {lim}\limits_{n \to \infty } \prod\limits_{i = 0}^{n} {\left[ {\alpha_{i} \left( {X_{i} ,X_{i + 1} ,\sigma_{i} } \right)} \right] = 0} .$$

Then, there exists a best approximation solution limit of (33) $X^{0} = lim_{n \to \infty } X_{n}^{0}$.

Assume, in addition that $$G = GG^{\dag } G$$, $$G^{\dag } = G^{\dag } GG^{\dag }$$, $$(GG^{{{\dag }}} )^{*} = GG^{*} ,$$$$(G^{{{\dag }}} G)^{*} = G^{*} G$$ and that {*G*_*n*_} → *G*, {*L*_*n*_} → *L* and {*B*_*n*_} → *B*. Then $$G^{{{\dag }}}$$ is unique and $$lim_{n \to \infty } X_{n}^{0} = X^{0} = (G - L)^{{{\dag }}} B = (I - G^{{{\dag }}} L)^{{^{{{\dag }}} }} B$$.

### *Proof*

Direct calculations from (33),$$G_{n}^{{{{\dag }}^{ *} }} = G_{n}^{{*^{{{\dag }}} }} ,$$$$B_{n} = \tilde{L}_{n} X_{n} ,$$$$Y_{n} = \tilde{M}_{n} X_{n}$$ and $$\Delta_{n} = (I - G_{n}^{\dag } G_{n} )Y_{n} = (I - G_{n}^{\dag } G_{n} )\tilde{M}_{n} X_{n}$$; $$\forall n \in \varvec{Z}_{0 + }$$ and $$lim\,sup_{n \to \infty } \left\| {H_{n} } \right\|_{2} < 1$$ yield:

39a $$\begin{aligned} &X_{n + 1}^{*} X_{n + 1} - X_{n}^{*} X_{n}\\ &\quad = - X_{n}^{*} \left[ {I - \left( {L_{n}^{*} G_{n}^{{*^{{{\dag }}} }} + G_{n}^{{{\dag }}} L_{n} } \right)} \right]X_{n}^{*} \\ & \quad\quad + \left( {B_{n}^{*} G_{n}^{{*^{{{\dag }}} }} + \Delta_{n}^{*} } \right)\left( {G_{n}^{{{\dag }}} B_{n} + \Delta_{n} } \right) + X_{n}^{*} L_{n}^{*} G_{n}^{{*^{{{\dag }}} }} \left( {G_{n}^{{{\dag }}} B_{n} + \Delta_{n} } \right) \\ &\quad \quad + \left( {B_{n}^{*} G_{n}^{{*^{{{\dag }}} }} + \Delta_{n}^{*} } \right)G_{n}^{{{\dag }}} L_{n} X_{n} \\ &\quad = - X_{n}^{*} \left[ {I - \left( {L_{n}^{*} G_{n}^{{*^{{{\dag }}} }} + G_{n}^{{{\dag }}} L_{n} } \right) - } \right.\left( {\tilde{L}_{n}^{*} G_{n}^{{*^{{{\dag }}} }} + \tilde{M}_{n}^{*} \left( {I - G_{n}^{*} G_{n}^{{*^{{{\dag }}} }} } \right)} \right)\left( {G_{n}^{{{\dag }}} \tilde{L}_{n} + \left( {I - G_{n}^{{{\dag }}} G_{n} } \right)\tilde{M}_{n} } \right) \\ \end{aligned}$$

39b $$\quad \left. { - L_{n}^{*} G_{n}^{{*^{\dag } }} \left( {G_{n}^{\dag } \tilde{L}_{n} + \left( {I - G_{n}^{\dag } G_{n} } \right)\tilde{M}_{n} } \right) - \left( {\tilde{L}_{n}^{*} G_{n}^{{*^{\dag } }} + \tilde{M}_{n}^{*} \left( {I - G_{n}^{*} G_{n}^{{*^{\dag } }} } \right)} \right)G_{n}^{\dag } L_{n} } \right]X_{n}$$

39c $$\quad = - X_{n}^{*} (I - H_{n} )X_{n} \le 0;\quad \forall n \in \varvec{Z}_{0 + }$$

from (39c) since*H*_*n*_ and $lim\,sup_{n \to \infty } (I - H_{n} )$ are symmetric semidefinite positive matrices; $\forall n \in \varvec{Z}_{0 + }$, then with no negative eigenvalues, since $0 \le lim\,sup_{n \to \infty } \left\| {H_{n} } \right\|_{2} \le 1$. If $0 \le \sum\nolimits_{i = 0}^{\infty } {\lambda_{j} (I - H_{n} ) < \infty }$ for $j \in \bar{q},$ with *λ*_*j*_(·) denoting some of the eigenvalues of the (·)-matrix, then for some $\{ Q_{n} \} \subset \varvec{C}^{q \times q} ,$, one has

40 $$\begin{aligned} X_{n + 1}^{*} X_{n + 1} - X_{m}^{*} X_{m} & = \sum\limits_{j = m}^{n} {\left( {X_{j + 1}^{*} X_{j + 1} - X_{j}^{*} X_{j} } \right)} \\ & = - \sum\limits_{j = m}^{n} {X_{j}^{*} (I - H_{j} )X_{j} \le - X_{n}^{*} \left( {\sum\limits_{j = m}^{n} {\left( {I - H_{j} } \right)X_{n} } } \right)} ;\quad \forall n \in \varvec{Z}_{0 + } \\ \end{aligned}$$

41 $$X_{n}^{*} Q_{n} X_{n} \le X_{n}^{*} \left( {\sum\limits_{j = 0}^{n} {I - H_{j} } } \right)X_{n} \le X_{0}^{*} X_{0} - X_{n + 1}^{*} X_{n + 1} \le X_{0}^{*} X_{0} ;\quad \forall n \in \varvec{Z}_{0 + }$$

Then, $\{ X_{n}^{*} X_{n} \} \subset \varvec{C}^{r \times r}$ converges to a positive (at least) semidefinite limit matrix $\bar{X}$ which can be factorized as $\bar{X} = \hat{X}^{*} \hat{X}.$. On the other hand, if $\{ \tilde{L}_{n} \} \to 0$ and $\{ \tilde{M}_{n} \} \to 0$ or if $\{ \tilde{L}_{n} \} \to 0$, and {*Y*_*n*_} → 0 without its generation from $Y_{n} = \tilde{M}_{n} X_{n} ,$ then $lim\,sup_{n \to \infty } \lambda_{\hbox{max} } (L_{n}^{*} G_{n}^{{*^{{{\dag }}} }} + G_{n}^{{{\dag }}} L_{n} ) < 1$. If $0 \le \sum\nolimits_{i = 0}^{\infty } {(I - L_{n}^{*} G_{n}^{{*^{{{\dag }}} }} + G_{n}^{{{\dag }}} L_{n} )} < \infty$, yields the same conclusion from (39a) and (39c). Now, $\{ X_{n}^{*} X_{n} \} \to \bar{X} = \hat{X}^{*} \hat{X}$ with $rank(\bar{X}) = rank(\hat{X}) = \ell \le min(q,r)$. Thus, there is a subsequence of matrices of rank ℓ,$\{ X_{{n_{k} }} \} \subset \{ X_{n} \} \subset \varvec{C}^{q \times r}$, such that $X_{{n_{k} + 1}} = \hat{U}_{{n_{k} }} X_{{n_{k} }}$ for some subsequence $\{ \hat{U}_{{n_{k} }} \} \subset \{ U_{n} \} \subset \varvec{C}^{q \times q}$ of non-singular matrices such that $rank\,X_{{n_{k} + 1}} = rank(\hat{U}_{{n_{k} }} X_{{n_{k} }} ) = rank\,X_{{n_{k} }} = rank\,\hat{X} = \ell$; $\forall n \in \varvec{Z}_{0 + }$. Then $X_{{n_{k} }}^{*} \hat{U}_{{n_{k} }}^{*} \hat{U}_{{n_{k} }} X_{{n_{k} }} \to \hat{X}^{*} \hat{X}$, and then $(\hat{U}_{{n_{k} }} X_{{n_{k} }} - \hat{X}) \to 0$ and $(X_{{n_{k} }} - \hat{U}_{{n_{k} }}^{ - 1} \hat{X}) \to 0$, as *k* → ∞and there exists a sequence $\{ U_{n} \}$ of nonsingular matrices such that $lim_{n \to \infty } (X_{n} - U_{n} \hat{X}) = 0$, $\forall n \in \varvec{Z}_{0 + }$. Property (i) has been proved.

To prove Property (ii), $X^{0} = (G - L)^{\dag } B$ is the best approximation solution nominal algebraic equation $GX = LX + B$.Thus, *GX* = *LX* + *B* is satisfied by *X*^0^ if and only if

42 $$G(G - L)^{\dag } B = \left( {I + L(G - L)^{\dag } } \right)B$$

(Moore 1935). Note that if (42) holds then direct calculations yield:

43 $$\begin{aligned} G^{{{\dag }}} (L - G)X^{0} + G^{{{\dag }}} B & = - G^{{{\dag }}} (G - L)(G - L)^{{{\dag }}} B + G^{{{\dag }}} B \\ & = G^{{{\dag }}} \left( {I - (G - L)(G - L)^{{{\dag }}} } \right)B \\ & = G^{{{\dag }}} B - G^{{{\dag }}} G(G - L)^{{{\dag }}} B + G^{{{\dag }}} L(G - L)^{{{\dag }}} B \\ & = - G^{{{\dag }}} G(G - L)^{{{\dag }}} B + G^{{{\dag }}} \left( {I + G^{{{\dag }}} L(G - L)^{{{\dag }}} } \right)B \\ & = - G^{{{\dag }}} G(G - L)^{{{\dag }}} B + G^{{{\dag }}} G(G - L)^{{{\dag }}}B\\ & = 0 \\ \end{aligned}$$

Rewrite the iterative scheme (31) in equivalent error form by defining the best approximation error solution related to $X^{0} \{ \tilde{X}_{n}^{0} \}$ by $\tilde{X}_{n}^{0} = X_{n}^{0} - X$; $\forall n \in \varvec{Z}_{0 + }$. This yields using (43):

44 $$\begin{aligned} \tilde{X}_{n + 1}^{0} & = G^{{{\dag }}} L + G^{{{\dag }}} \left( {(L - G)X^{0} + B} \right) \\ & = G^{{{\dag }}} L\tilde{X}_{n}^{0} + G^{{{\dag }}} \left( {I - (G - L)(G - L)^{{{\dag }}} } \right)B \\ & = G^{{{\dag }}} L \tilde{X}_{n}^{0} ;\quad \forall n \in \varvec{Z}_{0 + } \\ \end{aligned}$$

Now, if $\left\| {G^{{{\dag }}} L} \right\|_{2} < 1$ then $\{ \tilde{X}_{n}^{0} \} \to 0$ and $\{ X_{n}^{0} \} \to X^{0}$.

On the other hand, define an ordering in the best approximation real matrix solution as the entry-to-entry ordering of real numbers so that $\tilde{X}_{n + 1}^{0} \le \tilde{X}_{n}^{0}$ if and only if $(\tilde{X}_{n + 1}^{0} )_{ij} \le (\tilde{X}_{n}^{0} )_{ij}$. If now, *G* is surjective and inverse monotone and $- (G^{{{\dag }}} L)_{ij} \ge 0$ then $\tilde{X}_{n + 1}^{0} \le \tilde{X}_{n}^{0}$ if and only if $(\tilde{X}_{n + 1}^{0} )_{ij} \le (\tilde{X}_{n}^{0} )_{ij}$, provided that $(\tilde{X}_{0}^{0} )_{ij} \ge 0$, and $\tilde{X}_{n + 1}^{0} \ge \tilde{X}_{n}^{0}$, provided that $(\tilde{X}_{0}^{0} )_{ij} \le 0$, for each $n \in \varvec{Z}_{0 + }$. As a result, $\{ \tilde{X}_{n}^{0} \} \to 0$.Property (ii) has been proved.

To prove Property (iii), first note that {*G*_*n*_} → *G*, $$\{ L_{n} \} \to L$$ and $$\{ B_{n} \} \to B$$ imply that the sequences are bounded and that $$\left\| {G_{n} L_{n} } \right\|_{2} < 1$$ for *n* ≥ *n*_0_ and some $$n_{0} \in \varvec{Z}_{0 + }$$ since $$\left\| {G^{{^{{{\dag }}} }} L} \right\|_{2} < 1$$. As a result, $$lim\,sup_{n \to \infty } \sum\nolimits_{{i = n_{0} }}^{n} {\left\| {G_{i}^{{{\dag }}} L_{i} } \right\|_{2} } < \infty$$ and $$\{ \left\| {X_{n}^{0} } \right\|_{2} \}$$ is a bounded sequence, so the solution is bounded. Now, consider the incremental solution $$\tilde{X}_{n}^{0} = X_{n}^{0} - X^{0} ;$$$$\forall n \in \varvec{Z}_{0 + }$$. It satisfies the iterative scheme:

45 $$\tilde{X}_{n + 1}^{0} = G^{{^{{{\dag }}} }} \left( {L\tilde{X}_{n}^{0} + \left( {\tilde{B}_{n} + \tilde{L}_{n} X_{n}^{0} - \tilde{G}_{n} X_{n + 1}^{0} } \right) + \left( {LX - GX + B} \right)} \right);\quad \forall n \in \varvec{Z}_{0 + }$$

and

46 $$\begin{aligned} \left\| {\tilde{X}_{{n + 1}}^{0} } \right\|_{2} & \le \left\| {\prod\limits_{{i = 0}}^{{n + 1}} {\left[ {G^{\dag } L} \right]^{{^{i} }} X_{0} } } \right\|_{2} + \left\| {\sum\limits_{{i = 0}}^{n} {\left[ {G^{\dag } L} \right]^{{^{{n + 1 - i}} }} V_{n} } } \right\|_{2} \\ & \le \left\| {\prod\limits_{{i = 0}}^{{n + 1}} {\left[ {G^{\dag } L} \right]^{{^{i} }} X_{0} } } \right\|_{2} + \left\| {\sum\limits_{{i = 0}}^{\infty } {\left[ {G^{\dag } L} \right]} ^{{n + 1 - i}} V_{n} } \right\|_{2} \\ & \le K\rho ^{{n + 1}} \left\| {X_{0} } \right\|_{2} + \frac{{K\delta }}{{1 - \rho }} \\ \end{aligned}$$

47 $$\mathop {lim\,sup}\limits_{n \to \infty } \left\| {\tilde{X}_{n + 1}^{0} } \right\|_{2} \le \mathop {lim\,sup}\limits_{n \to \infty } \left\| {\sum\limits_{i = 0}^{n} {\left[ {G^{{^{\dag } }} L} \right]^{{^{n + 1 - i} }} V} } \right\|_{2} \le \frac{\delta }{1 - \rho }$$

for some $$K \in \varvec{R}_{ + }$$ and *ρ* $$\in$$ (0, 1), since $$LX - GX + B = 0,$$ and $$\left\| {X_{n}^{0} } \right\|_{2} ,$$ {*G*_*n*_}, {*L*_*n*_}, {*B*_*n*_} and then {*V*_*n*_}, with $$V_{n} = \tilde{B}_{n} + \tilde{L}_{n} X_{n}^{0} - \tilde{G}_{n} X_{n + 1}^{0} ;$$$$\forall n \in \varvec{Z}_{0 + }$$, are bounded. Property (iii) has been proved.

To prove Property (iv), define $$M(X_{n + 1}^{0} ,X_{n}^{0} ,t) = \frac{t}{{t + \left\| {X_{n + 1}^{0} - X_{n}^{0} } \right\|_{2} }}$$; $$\forall n \in \varvec{Z}_{0 + }$$, $$\forall t \in \varvec{R}_{ + }$$. From the contractive condition (11), one has $$M(X_{n + 2}^{0} ,X_{n + 1}^{0} ,t) \ge M(X_{n + 1}^{0} ,X_{n}^{0} ,\alpha_{n}^{ - 1} t);$$$$\forall n \in \varvec{Z}_{0 + }$$, $$\forall t \in \varvec{R}_{ + }$$ with strict inequality if $$M(X_{n + 1}^{0} ,X_{n}^{0} ,\alpha_{n}^{ - 1} t) < 1$$. Since $$rank\,G_{n} = s;$$$$\forall n \in \varvec{Z}_{0 + }$$ the mapping $$g:\varvec{C}^{q \times r} \to \varvec{C}^{s \times r}$$ is surjective. Then, such an inequality holds for all $$t \in \varvec{R}_{ + }$$ from (39c) if for some sequence $$\{ m_{n} \} \subset \varvec{Z}_{ + }$$:

48 $$\alpha_{n} \ge \frac{{\left( {\left( {\left\| {\left( {G_{n + 1}^{{{\dag }}} L_{n + 1} - I} \right)\left( {G_{n}^{{{\dag }}} \left( {L_{n} X_{n}^{0} + B_{n} } \right)} \right) + G_{n + 1}^{{{\dag }}} B_{n + 1} } \right\|_{2} + t} \right)t^{{ - m_{n} }} - 1} \right)t}}{{\left\| {\left( {G_{n}^{{{\dag }}} L_{n} - I} \right)X_{n}^{0} + G_{n}^{{{\dag }}} B_{n} } \right\|_{2} }};\quad \forall n \in \varvec{Z}_{0 + } ,\quad t \in \varvec{R}_{ + }$$

Thus, for any switching law $\sigma :\varvec{R}^{2} \times \varvec{Z}_{0 + } \to \bar{p}$, with a growth evolution rule $\alpha_{n} = \alpha (X_{n + 1}^{0} ,X_{n}^{0} ,\sigma (X_{n + 1}^{0} ,X_{n}^{0} ,{\mathbf{Z}}_{0 + } )) \in \varOmega$ and functions $\psi_{{\sigma_{n} }} \in \varPsi (\sigma ,\alpha )$, such that $lim_{n \to \infty } \prod\nolimits_{i = 0}^{n} {[\alpha_{i} (X_{i}^{0} ,X_{i + 1}^{0} ,\sigma_{i} (X_{i}^{0} ,X_{i + 1}^{0} ))]} = 0$, it follows that there exists a best approximation solution limit of (33) $X^{0} = lim_{n \to \infty } X_{n}^{0}$.

On the other hand, if {*G*_*n*_} → *G*, {*L*_*n*_} → *L* and {*B*_*n*_} → *B* then $$\{ G_{n}^{{{\dag }}} L_{n} \}$$ and $$\{ G_{n}^{{{\dag }}} B_{n} \}$$ converge. If $$rank\,G_{n} = s$$; $$\forall n \in \varvec{Z}_{0 + }$$ and, furthermore, (37) and (38) hold, then {*X*_*n*_} converges. If *X*^0^ is the best approximation solution of the limit system and if $$G = GG^{{{\dag }}} G,$$$$G^{\dag } = G^{\dag } GG^{\dag } ,$$$$(GG^{{{\dag }}} )^{*} = GG^{*}$$, $$(G^{{{\dag }}} G)^{*} = G^{*} G$$, then $$G^{{{\dag }}}$$ is unique (Barnett 1971), and thus $$X^{0} = (G - L)^{{{\dag }}} B = (I - G^{{{\dag }}} L)^{{{\dag }}} G^{{{\dag }}} B$$ is the best approximation limit solution (see also Property (ii)). Define the solution error with respect to the best approximated solution in the constant case as $$\tilde{X}_{n}^{0} = X_{n}^{0} - X^{0} ;$$$$\forall n \in \varvec{Z}_{0 + }$$. Thus:

49 $$\begin{aligned} \tilde{X}_{n + 1}^{0} & = G_{n}^{{{\dag }}} L_{n} X_{n}^{0} + \left( {G_{n}^{{{\dag }}} B_{n} - X^{0} } \right);\quad \forall n \in \varvec{Z}_{0 + } \\ & = G_{n}^{{{\dag }}} L_{n} \tilde{X}_{n}^{0} + \left( {G_{n}^{{{\dag }}} B_{n} + \left( {G_{n}^{{{\dag }}} L_{n} - I} \right)X^{0} } \right);\quad \forall n \in \varvec{Z}_{0 + } \\ \end{aligned}$$

50 $$\mathop {lim\;sup}\limits_{n \to \infty } \left( {\tilde{X}_{n + 1}^{0} - G_{n}^{{{\dag }}} L_{n} X_{n}^{0} } \right) = \mathop {lim\;sup}\limits_{n \to \infty } \left( {G_{n}^{{{\dag }}} B_{n} - X^{0} } \right) = E$$

51 $$\mathop {lim\;sup}\limits_{n \to \infty } \left( {\tilde{X}_{n + 1}^{0} - G_{n}^{{{\dag }}} L_{n} \tilde{X}_{n}^{0} } \right) = \mathop {lim\;sup}\limits_{n \to \infty } \left( {G_{n}^{{{\dag }}} B_{n} + \left( {G_{n}^{{{\dag }}} L_{n} - I} \right)X_{0} } \right) = 0$$

and $lim_{n \to \infty } \tilde{X}_{n}^{0} = 0$, since $lim\,sup_{n \to \infty } \left\| {G_{n}^{{{\dag }}} L_{n} } \right\|_{{_{2} }} < 1$, and then $lim_{n \to \infty } X_{n}^{0} = X^{0} .$ □

## Conclusions

The existence, uniqueness and limit properties for proximal sequences of optimal fuzzy best proximity coincidence points have been investigated for pairs of mappings defining proximal sequences $\{ (g_{n} ,T_{n} )\}$, with $g:A \to A$ being surjective and $T:A \to B$, where *A* and *B*are nonempty subsets of a nonempty set *X*. The best proximity coincidence points are defined in partially ordered non-Archimedean fuzzy metric spaces $(X,M, * ,\underline{ \prec } )$ for so-called fuzzy order preserving proximal *Ψ*(*σ*, *α*)-lower-bounding mappings where * is a triangular norm, $\underline{ \prec }$ is an ordering relation and *M*is a fuzzy set which evolves according to a switching rule $\{ \sigma_{n} \} \subset \varvec{Z}_{ + }$. The concerned fuzzy sets have been interpreted, in the general case, as sequences defined depending of the switching rules. Also, three application examples are described concerned with: (a) switched stabilization of fuzzy discrete dynamic systems, and (b) best approximations of resolution of equations in linear algebra. Some “ad hoc” specific related results for the examples are also given and proved. The proposed formalism seems to be also of usefulness for extensions of studies of stability and stabilization and for proximal approaches in the fuzzy framework.
